# IGLR-2, a Leucine-Rich Repeat Domain Containing Protein, Is Required for the Host Defense in *Caenorhabditis elegans*

**DOI:** 10.3389/fimmu.2020.561337

**Published:** 2020-11-30

**Authors:** Cheng-Ju Kuo, Ya-Chu Hsu, Sin-Tian Wang, Bang-Yu Liou, Serene Boon-Yuean Lim, Yi-Wei Chen, Chang-Shi Chen

**Affiliations:** ^1^ Department of Biochemistry and Molecular Biology, College of Medicine, National Cheng Kung University, Tainan, Taiwan; ^2^ Institute of Basic Medical Sciences, College of Medicine, National Cheng Kung University, Tainan, Taiwan

**Keywords:** *Caenorhabditis elegans*, *iglr-2*, innate immunity, p38 MAPK pathway, enterohemorrhagic *Escherichia coli*

## Abstract

Enterohemorrhagic *Escherichia coli* (EHEC), a human pathogen, also infects *Caenorhabditis elegans*. We demonstrated previously that *C. elegans* activates the p38 MAPK innate immune pathway to defend against EHEC infection. However, whether a *C. elegans* pattern recognition receptor (PRR) exists to regulate the immune pathway remains unknown. PRRs identified in other metazoans contain several conserved domains, including the leucine-rich repeat (LRR). By screening a focused RNAi library, we identified the IGLR-2, a transmembrane protein containing the LRR domain, as a potential immune regulator in *C. elegans*. Our data showed that *iglr-2* regulates the host susceptibility to EHEC infection. Moreover, *iglr-2* is required for pathogen avoidance to EHEC. The *iglr-2* overexpressed strain, which was more resistant to EHEC originally, showed hypersusceptibility to EHEC upon knockdown of the p38 MAPK pathway. Together, our data suggested that *iglr-2* plays an important role in *C. elegans* to defend EHEC by regulating pathogen-avoidance behavior and the p38 MAPK pathway.

## Introduction

Animal hosts constantly encounter a variety of pathogenic microorganisms. Appropriate sensing of pathogenic microbes and an effective response to infection are crucial for their survival. The receptors through which metazoans detect pathogen/microbe-associated molecular patterns (PAMPs/MAMPs) or damage-associated molecular patterns (DAMPs) are called pattern recognition receptors (PRRs) (1, 2). PRRs mediate the launch of innate immune response resulting in antimicrobial peptide expression while recognizing PAMPs or DAMPs (2). This process is called pattern-triggered immunity (PTI) (1). Intriguingly, no related receptors responsible for pathogen sensing have yet been identified in *C. elegans*. Although the receptors that function upstream of the immunity pathway remain unknown, several pieces of evidence imply that PTI exists in *C. elegans.* It has been reported that the intact LPS of *Salmonella* *enterica* is required for the induction of p38 MAPK immunity pathway during infection in *C. elegans* (3, 4). This suggested that the upstream receptors detect bacterial LPS to activate immune response. *Pseudomonas aeruginosa* secretes toxins to kill *C. elegans*. However, a study showed the secreted factors of *P. aeruginosa* in medium did not induce an immune response, but exposure to a non-pathogenic *Pseudomonas mendocina* did upregulate several specific immune-related genes (5). In addition, nematodes were found to induce 56% of identical genes (*e.g.*, antifungal genes) while infected with either live or heat-killed *Candida albicans via* transcriptomic analysis (6). Congruent with these results, heat-killed *Staphylococcus aureus* stimulated a similar immune response as live *S. aureus* to *C. elegans* (7). These studies indicate that *C. elegans* detects pathogens by targeting not only toxins but also some pathogenic components. *C. elegans* can also trigger distinct host defense responses while facing different pathogenic insults (7–9). Moreover, recent reports also demonstrated that *C. elegans* can sense intracellular DNA and RNA to activate a DAMP and PAMP response (10, 11). These lines of evidence suggest that *C. elegans* might have unique receptors for sensing either pathogens or their products to mount PAMP or DAMP responses.

PRRs from different species share specific evolutionarily conserved motifs or domains, which are usually unique to microbes for recognizing conserved molecular structures of pathogenic microbes (*e.g.*, bacterial flagellin, bacterial LPS or viral ssRNA). The most well-known pathogen recognition domain is the leucine-rich repeat (LRR) domain. The toll-like receptor (TLR) family, the NOD-like receptor family (NLR) in animals and the leucine-rich repeat receptor-like kinase (LRR-RLK) in plants contain LRR domains. TLRs were first discovered in *Drosophila melanogaster* to recognize microbial patterns as well as the endogenous ligand, Spatzle [or Spaetzle, Spätzle (Spz)] triggering the host defense response and have since been found in many other species (12). In humans, TLRs are located on the cell membrane or in the endosome where they sense specific PAMPs/MAMPs to activate the nuclear factor-*κ*B (NF*κ*B) or the p38 mitogen-activated protein kinase (MAPK) pathway (2). The LRR domain at the N-terminal tail of TLRs is responsible for the recognition of extracellular or endosomal ligands (13). Once the ligands bind to receptors, TLRs become dimerized and trigger downstream signaling activation (*e.g.*, NF-*k*B, IRF-7, or p38 MAPK pathway) (13). For example, while bacterial lipopolysaccharide (LPS) binds to Toll-like receptor 4, Toll/Interleukin-1 receptor homology (TIR) domain in the cytoplasm recruits adaptor protein, MyD88, to activate NF-kB resulting in proinflammatory cytokine expression *via* phosphorylation cascade (14). The nucleotide-binding and oligomerization domain (NOD)-like receptor family (NLR) are the receptors located in the cytoplasm, containing NOD domain in the central, signal domain at the N-terminus and an LRR domain at the C-terminus which is involved in sensing and binding to ligands (15). Two classical NLRs, NOD1 and NOD2 receptors, are essential for recognizing diaminopimelic acid and muramyl dipeptide (both are bacterial peptidoglycan derivatives), respectively. The recognition stimulates NF-*κ*B, interferon (IFN) regulatory factors (IRFs) and consequently MAPK pathway activation (16). Leucine-rich repeat receptor-like kinases (LRR-RLK), which are well-characterized in plants, contain an extracellular LRR domain, a transmembrane region and a cytoplasmic Ser/Thr protein kinase domain at the C-terminus (17). In *Arabidopsis thaliana*, bacterial flagellin binding to FLAGELLIN-SENSING 2 (FLS2), a LRR-RLK, triggers MAPK by phosphorylation cascades allowing initiation of intracellular signaling to activate antimicrobial genes and generate ROS to defend pathogen infection (18).

Our previous research demonstrated that EHEC infects *C. elegans* and also activates the host p38 MAPK pathway (19). However, how *C. elegans* perceives EHEC bacteria to activate the innate immune response *per se* remains elusive. Here, we identified a gene, *iglr-2*, that containing immunoglobulin-like, LRR and transmembrane domains is required for host defense by screening a PRR focused RNAi library. We then generated *iglr-2* null mutants using CRISPR-Cas9 method and found that *iglr-2* mutants were hypersensitive to EHEC and were not able to avoid EHEC Infection. Moreover, the enhanced survival of *iglr-2* overexpressing strains to EHEC killing was suppressed by p38 MAPK pathway RNAi, and the hypersensitive phenotype of *iglr-2* null mutant was restored to wild type by the constitutive activation of p38 MAPK cascades in *iglr-2* mutant. Our genetic data suggested that *iglr-2* is involved in EHEC bacteria avoidance behavior and the p38 MAPK pathway acts downstream of *iglr-2*, partly, in *C. elegans*.

## Materials and Methods

### Bacterial and Nematode Strains

The nematode strains used in this study are listed in **Table S1**. The *E. coli* O157:H7 clinical isolates strain EDL933 were from the Bioresource Collection and Research Center (BCRC), Taiwan. *Caenorhabditis elegans* strains TU3401, VP303, WM118, and SAL144 used in this work were kindly provided by the *Caenorhabditis* Genetics Center (CGC), which is funded by the NIH National Center for Research Resources (NCRR). *iglr-2* deletion strains were generated by the CRISPR-Cas9 system as described (20, 21). *iglr-2* overexpressed strains, transcriptional and translational reporter strains were created by microinjection as described (22, 23). The genotype of the above *C. elegans* strains were confirmed by single worm PCR, restriction fragment length polymorphism, and sequencing analysis. *C. elegans* strains were maintained on nematode growth medium (NGM) agar plates using the standard laboratory *E. coli* strain OP50 as described (24).

### PRR RNA Library Construction and Screening

Several categories of genes are reported to contain specific motifs or domains considered as PRRs or potential PRRs (2) and hence were selected into our RNAi library *e.g*., C-type lectin-like domains (CTLDs), galectin, lipid binding protein (LBP), leucine-rich repeat domain (LRR), lysin motif domain (LysM), and peptidoglycan recognition protein (PGRP). We utilized WormMart on WormBase to identify the genes that contained domains of LRR, LBP, LysM, galectin, or PGRP. WormMart was terminated in 2016; however, a similar function can be found on BioMart. The study by Schulenburg et al. was used as a reference for genes containing C-type lectin-domains (25). RNAi clones are collected from the Ahringer RNAi or constructed from the Worm ORFeome library *via* Gateway cloning system. The constructed PRR library was frozen in a 96-well format with 8% glycerol (Sigma-Aldrich) at −80°C before use. The detailed screening method is as described and illustrated in the Supplemental Information (**Figure S1**). The RNAi clones and the results of screening are listed in **Table S2**.

### Survival Analysis

All survival assays were performed as previously described (19, 26, 27). Briefly, *E. coli* O157:H7 EDL933 bacteria were cultured in Luria–Bertani (LB) broth for 16 to 18 h at 37°C. A 30 µL aliquot of bacterial culture (O.D.600 nm = 2) was plated on 5.0 cm nematode growth media (NGM) agar plates and incubated overnight at room temperature (25°C). Approximately fifty synchronized late larval stage 4 (L4) to young adult animals were transferred to each plate and maintained at 20°C. Worms were scored daily and considered as dead when animals did not respond to touch by platinum wire. Animals that crawled off the plate were scored as censored. Worms were transferred to fresh plates every day throughout the reproductive period. The assay was conducted independently at least three times with approximately 50 to 100 worms each time at 20°C. Data represent the sum of animals in multiple experiments in the figures. The number of independent survival assays and sample sizes for each of these experiments is given in **Table S3**.

### Lawn Occupancy Assays

The assay was conducted as previously described with slight modifications (28, 29). Bacterial (*E. coli* OP50, *E. coli* O157:H7 EDL933 or *P. aeruginosa* PA14) lawns were prepared by inoculating individual bacterial colonies into 3 mL of Luria–Bertani (LB) broth and grown for 16 to 18 h in a 37°C incubator. A 30 µL aliquot of bacterial culture was plated onto the center of a 10 cm NGM plate and kept at room temperature (25°C) for 24 h. Approximately thirty synchronized late larval stage 4 (L4) animal worms were transferred onto plates’ center containing the bacterial lawn and scored after 16 h incubation at 20°C. Each animal was scored as inside or outside the lawn (Occupancy Index = N in/N total). Experiments were performed for at least three times independently.

### CRISPR-Cas9 System for Targeted Genome Editing

For CRISPR disruption of *iglr-2*, we used the technique previously described (20, 21) using the plasmids, pDD162 (21) (Peft-3::Cas9 + Empty sgRNA) (plasmid number 47549 on Addgene) and PU6::unc-119_sgRNA (21) (plasmid number 46169 on Addgene). Briefly, sgRNA targeting the 6^th^ and 10^th^ exons of *iglr-2* was created by using overlap extension PCR and TA cloning system. We used PU6::unc-119_sgRNA as a template, U6prom *EcoRI* F (5′-CGG GAA TTC CTC CAA GAA CTC GTA CAA AAA TGC TCT-3′), *iglr-2* (a + b) gRNA R (5′-TTA GAT ATC GAG CAG AGA ACA AAC ATT TAG ATT TGC AAT TCA ATT ATA TAG), *iglr-2* (a + b) gRNA F (5′-GTT CTC TGC TCG ATA TCT AAG TTT TAG AGC TAG AAA TAG CAA GTT A-3′) and U6prom *HindIII* R (5′-CGG AAG CTT CAC AGC CGA CTA TGT TTG GCG T-3′) to construct sgRNA targeting the 6^th^ exon. As above, we used U6prom *EcoRI* F (5′-CGG GAA TTC CTC CAA GAA CTC GTA CAA AAA TGC TCT-3′), *iglr-2* cytosol sgRNA R (5′-TTG AAT CCA CCA AAG AGC ACA AAC ATT TAG ATT TGC AAT TCAA TTA TAT AG-3′), *iglr-2* cytosol sgRNA F (5′-GTG CTC TTT GGT GGA TTC AAG TTT TAG AGC TAG AAA TAG CAA GTT A-3′) and U6prom *HindIII* R (5′-CGG AAG CTT CAC AGC CGA CTA TGT TTG GCG T-3′) to construct sgRNA targeting the 10^th^ exon. N2 animals were injected with about 150 ng/μL of total DNA, and the ratio of pDD162 to sgRNA to marker pBCN40-R4R3 (plasmid number 34915 on Addgene) was 10:9:1, respectively. Transgenic F1 progeny was screened for mCherry expression and plated individually. After F2 progeny production, F1 animals were screened by single worm PCR and restriction enzyme digestion to identify *iglr-2* homozygote mutants.

### *iglr-2* Transgenic Strain Generation

To analyze transcription of *iglr-2*, we PCR amplified the 218 bp region between the start codon of *iglr-2* and the 3′ end of the adjacent upstream gene from genomic DNA of wild-type animals. The primers 5′-GGGGACAACTTTGTATAGAAAAGTTGAATTGTTAGATTTTATTTAGATATTATCCTT-3′ and 5′-GGGGACTGCTTTTTTGTACAAACTTGTTCTTCTTTTCTTTGTATCAAGACA-3′ were used for amplifying the region. This promoter region was constructed to plasmid pCG144 (plasmid number 21376 on Addgene) and pCM5.37 (plasmid number 17253 on Addgene) to destination plasmid pCG150 (plasmid number 17247 on Addgene) generating pWF274 [*iglr-2p*::*mCherry*::*unc-54* 3′UTR] through the Gateway cloning system. Plasmid, pWF237 [*iglr-2p*::*mCherry*::histone H2B::*unc-54* 3′UTR], was constructed using the same method except that mCherry::histone H2B was from pCM1.151 (plasmid number 21386 on Addgene). To analyze the expression of IGLR-2 protein, we PCR-amplified full-length, wild-type *iglr-2* coding sequence isoform a (2,322 bp) and isoform b (1,875 bp) from cDNA, respectively and ligated it into the destination plasmid pCG150 *via* a Gateway cloning method to generate pWF294 [*iglr-2p*::*iglr-2a*::*mCherry*::*unc-54 3*′UTR] and pWF304 [*iglr-2p*::*iglr-2b*::*mCherry*::*unc-54 3*′UTR]. The primers (5′-GGGGACAAGTTTGTACAAAAAAGCAGGCTATGCGAAAATTTGTATTTTTCGTCGTAG-3′) (5′-ACCCTTTGAGACAGCAGCAGCAGCAGCAGCTCTCTTTTCTGGTGGAGAATCTG-3′) and the primers (5′-GGGGACAAGTTTGTACAAAAAAGCAGGCTATGCGAAAATTTGTATTTTTCGTCGTAG-3′) (5′-ACCCTTTGAGACAGCAGCAGCAGCAGCAGCTTCCTGAAGATGGTAAGTAAGTG-3′) were used for amplifying isoform a and isoform b, respectively.

### Quantitative RT-PCR

Assays were carried out as previously described (19). Synchronized populations of wild-type N2 and *iglr-2* overexpressed strain animals were grown to the late L4 larval stage and fed with *E. coli* OP50 or EDL933 for 12 and 24 h at 20°C. Total RNA was then extracted using TRI Reagent (Invitrogen) and reverse transcribed with M-MLV reverse transcriptase (Promega) using random hexamer primers. This cDNA was then subjected to qRT-PCR analysis using SYBR green detection on a 7,500 fast real-time PCR system (Applied Biosystems). Relative expression between samples was normalized to the *nhr-23* as the reference gene. The primers (5′-GCCGAAGATGATGCCGAGAT-3′; 5′-GTCGCA GTGTCAAGAATCCC-3′) were used for *nhr-23* gene detection, and the primers (5′-TTCTCC GAGATGGTGATTTGG-3′; 5′-TTCTCCGAGATGGTGATTTGG-3′) were used for *iglr-2* gene detection. All experiments were performed a minimum of three times independently.

### RNA Interference Plate Assay

RNAi experiments were performed as described with some modifications (30). In brief, RNAi *E. coli* strains HT115 transformed with RNAi plasmids were spread on NG-IC plates which are NGM plates with 50 µg/mL carbenicillin and 1 mM isopropyl-*β*-D-1-thiogalactopyranoside (IPTG). Plates with RNAi bacteria were incubated at 25°C overnight to induce the double-stranded RNA (dsRNA) expression. Synchronized wild-type N2, RNAi-sensitized strain *rrf-3(pk1426)*, and tissue specific RNAi knockdown strains L1 stage larvae were obtained using standard alkaline hypochlorite protocols (24). L1 stage larvae cultured on L4440 or plates expressing RNAi of interest at 20°C until F1 progenies grown to L4 stage. Survival assays were conducted by mixing control OP50-GFP or EDL933-GFP (19) (which were carrying an ampicillin resistant plasmid) with RNAi bacteria of 1:1 ratio. Mixed bacteria were spread on NGIC plates (30 µL per plate). Approximately fifty worms were picked to the prepared OP50/EHEC-RNAi plates and scored their survival as mentioned above. *E. coli* HT115 with L4440, an empty vector, was used as a negative control of RNAi. All experiments were performed a minimum of three times independently. Data represent the sum of animals in multiple experiments in the figures.

### Images of Nematodes

Late L4 to young adult stage of *iglr-2* reporter strains were plated on NGM plates with *E. coli* OP50 at 20°C for 12 h. For *K08D8.5* GFP expression analysis, late L4 to young adult stage of *C. elegans* strains with *K08D8.5::GFP* transgene in wild-type and *iglr-2(wf275)* background were infected with EHEC at 25°C for 8 h, respectively. Animals were then mounted on glass slides with 2% agarose pads and anesthetized with 25 nM sodium azide (NaN_3_). The fluorescent signal in the nematode was observed by differential interference contrast (DIC) imaging with Nomarski optics and epifluorescence imaging with corresponding filters using a Nikon Eclipse Ti inverted microscope system with DP72 CCD camera. The quantification of *K08D8.5::GFP* signal was measured by imageJ.

### Generation of Double Mutant in *iglr-2* Mutant Background

To generate *nsy-1(ums8)*;*iglr-2(wf295)* double mutant, wild-type N2 male animals were crossed to *iglr-2(wf295)* hermaphroditic gravid adults. Then, male animals of crossed *iglr-2* heterozygotes were picked to mate *nsy-1(ums8)* hermaphrodites. Their progenies of F2 generation were confirmed for *iglr-2(wf295), nsy-1(ums8)* or *nsy-1(ums8);iglr-2(wf295)* by single worm PCR and restriction fragment length polymorphism. The animal exhibited wild-type genotype of *iglr-2(wf295)*, and *nsy-1(ums8)* was selected as a control. *iglr-2(wf295)* was confirmed *via* using the primers (5′-TCTGAATGGAACTGTAATCC-3′; 5′-ATTATCGCGAAGAAT TATGA-3′), and the amplicon was then digested by *EcoRV* enzyme. *nsy-1(ums8)* was confirmed *via* using the primers (5′-CGATTTTGACAAATTGGACT-3′; 5′-AGACAAACAACG TCTGGACT-3′), and the amplicon was then digested by *HpyCH4V* enzyme. *nsy-1(ums8)* gain-of-function allele was a kind gift from Dr. Pukkila-Worley (31). To generate *iglr-2(wf275);K08D8.5::GFP*, male animals of SAL144 (32) were mated to *iglr-2(wf275)* hermaphroditic gravid adults. Their progenies of F2 generation showed GFP expression were isolated and confirmed for *iglr-2(wf275)* by the method as described above. The animal exhibited wild-type genotype of *iglr-2(wf275)* was selected as a control. The worm strain, SAL144 *pha-1(e2123);denEx22 [K08D8.5::GFP + pha-1(+)]* was a kind gift from Dr. Schwartz (32).

### EHEC Infection Assay of RPW-24 Treatment

The assay was performed as described (33) with slight modifications. Briefly, the RPW-24 agar plates were prepared by adding 5 µM of RPW-24 (MCE, catalog number: HY-W035409) compounds to 3.5 cm plates containing of 4 mL NGM agar. 10 µL overnight cultured EHEC bacteria were seeded to these RPW-24 agar plates and incubated for 24 h at 25°C. Then, thirty to thirty-five synchronized L4 to young adult staged *iglr-2* mutants were placed to RPW-24 agar plates and maintained at 20°C. NGM agar plates supplemented with 0.5% DMSO were used as negative controls. Animals were scored daily and considered as dead when nematodes did not respond to touch by platinum wire. Worms that crawled off the plate were scored as censored and were transferred to plates every day throughout the reproductive period. The assay was performed independently for four times.

## Results

### RNAi Screening for a Potential *C. elegans* PRR in EHEC Infection

**Figure 1:**
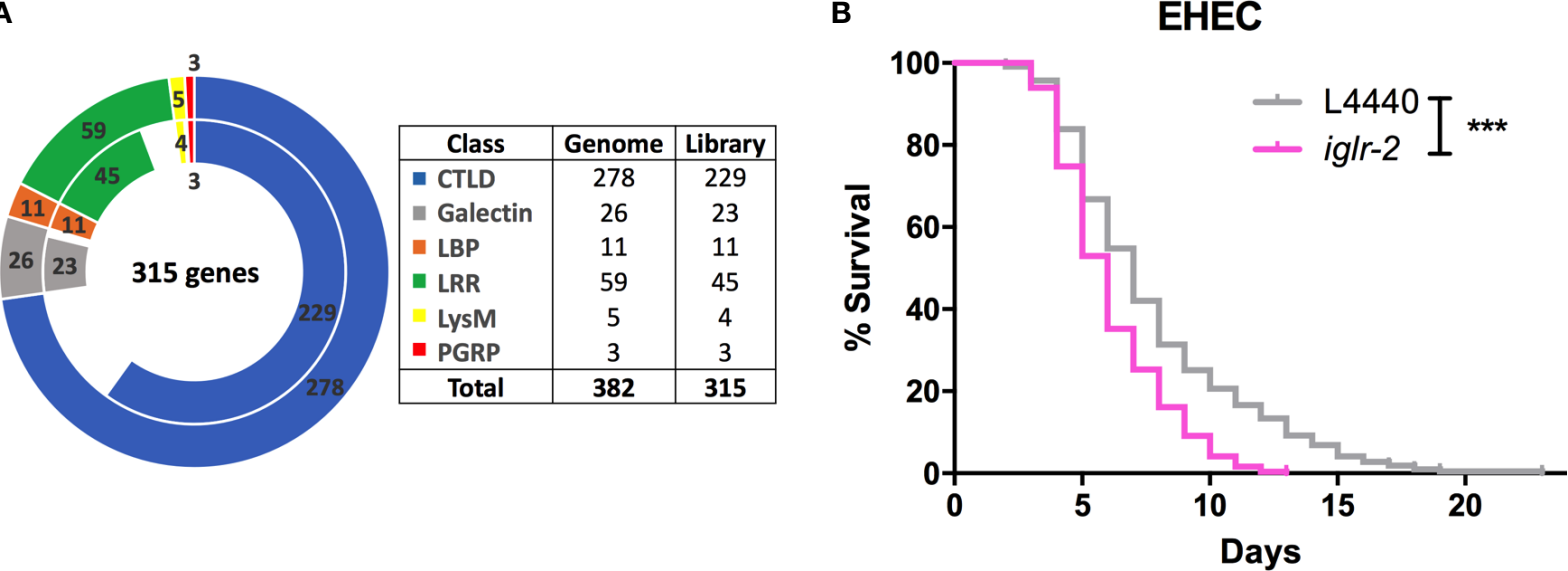
RNAi screening identified *iglr-2* is required for host defense in *C. elegans*. **(A)** The doughnut chart represents the number of selected genes containing specific proteins/domains that are considered as PRRs and the number of RNAi bacteria clones in RNAi library constructed from Ahringer RNAi library or Worm ORFeome library. The outer ring represents 382 selected genes considered as potential PRRs. The inner ring represents 315 RNAi clones. Sixty-seven genes are deficient in both Ahringer RNAi library or Worm ORFeome library. CTLDs, C-type lectin-like domains; LBP, Lipid binding protein; LRR, Leucine-rich repeat domain; LysM, Lysin motif domain; PGRP, Peptidoglycan recognition protein. **(B)** Knockdown of *iglr-2* by RNAi silencing resulted in N2 animals hypersensitive to *E. coli* O157:H7 strain EDL933 infection compared to that of L4440 (*P* < 0.001). ***P < 0.001 by the Mantel–Cox log-rank test. Survival curves represent the sum of animals in multiple experiments.

**Figure 2:**
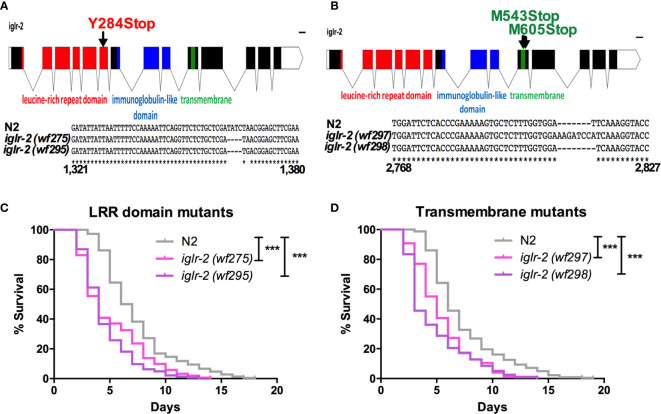
*iglr-2* null mutants created by CRISPR-Cas9 genome editing are hypersusceptible to EHEC. **(A, B)** Top: Diagram of *iglr-2* isoform transcript. Position and mutation as indicated. Colored filled boxes are exons. Boxes in red indicate the leucine-rich repeat (LRR) domain region. Boxes in blue indicate immunoglobulin-like (Ig-like) domains region. Boxes in green indicate the transmembrane region. Lines are introns. Blank regions are untranslated. ****Bottom: DNA sequence of *iglr-2* mutants compared to N2 wild type. **(C)** Mutation at LRR domain of *iglr-2* contributed *C. elegans* hypersusceptible to *E. coli* O157:H7 strain EDL933 infection. [N2 vs *iglr-2 (wf275)* *P* < 0.001, N2 vs *iglr-2* *(wf295)* *P* < 0.001]. **(D)** Survival curves of two *iglr-2* transmembrane domain mutants fed with EHEC exhibited a significant sensitive phenotype compared to that of N2 [*iglr-2 (wf297)* *P* < 0.001, *iglr-2 (wf298)* *P* < 0.001] animals at 20°C. ***P < 0.001 by the Mantel–Cox log-rank test. Survival curves represent the sum of animals in multiple experiments.

To identify potential pattern recognition receptors (PRRs) in *C. elegans*, we constructed and screened a PRR focused RNAi library. These RNAi clones target 315 genes that encode various PRRs specific motifs or domains (**Figure 1A** and **Table S2**). We identified that knockdown of seven genes, including *lron-9* (*P* < 0.05), *iglr-2* (*P* < 0.001), *mog-2* (*P* < 0.05), *sds-22* (*P* < 0.05), *soc-2* (*P* < 0.05), *pxn-2* (*P* < 0.05), *zmp-2* (*P* < 0.001), increased susceptibility of animals to EHEC infection when compared to the empty vector, L4440 by one-way ANOVA test. Among them, only *iglr-2* RNAi exhibited a significantly decreased survival of EHEC to *C. elegans* and did not cause developmental defect to compromise our analysis in the adult animals; we therefore turned our focus to analyze the role of *iglr-2* during EHEC infection in *C. elegans*. IGLR-2 is a transmembrane protein with an extracellular leucine-rich repeat domain at the N-terminus, an immunoglobulin-like domain in the center, and a transmembrane domain and an intracellular domain at the C-terminus (**Figure 2A**). To reconfirm our result from RNAi liquid-toxicity assay, we performed EHEC plate-toxicity assay with RNAi of *iglr-2*. Indeed, RNAi-mediated knockdown of *iglr-2* that targets the leucine-rich repeat (LRR) and immunoglobulin (IG)-like domains in wild-type N2 strain exhibited hypersusceptibility to EHEC (**Figure 1B**, *P* < 0.001 by Log-rank test).

### Loss of *iglr-2* in *C. elegans* Confers Hypersusceptibility to EHEC Infection

To strengthen our hypothesis that *iglr-2* is required for *C. elegans* to defend against EHEC O157:H7 strain EDL933 infection, we generated *iglr-2* deletion mutants by the clustered regularly interspaced short palindromic repeats and CRISPR-associated protein 9 (CRISPR-Cas9) genome-editing technology (20, 21). We designed single guide RNA (sgRNA) targeting at the LRR domain of *iglr-2* and generated two independent mutants (**Figure 2A**). *iglr-2(wf275)* mutant had four nucleotides deleted causing a tyrosine to change to a stop codon in codon 284 of the LRR domain by prediction of amino acid sequences. *iglr-2(wf295)* mutant not only had four nucleotides deleted; it also had one nucleotide changed (Adenosine to Guanosine) and a leading tyrosine changed to a stop codon in codon 284 of the LRR domain as well (**Figure 2A**). Furthermore, we also designed sgRNA targeting at the transmembrane domain of *iglr-2* and generated two independent mutants *iglr-2(wf297)* had one nucleotide alteration (Thymidine to Adenosine) and seven nucleotide insertion causing a start codon (Methionine) to change to a stop codon in codon 543 of the transmembrane domain by prediction of amino acid sequences. *iglr-2(wf298)* had one nucleotide deleted also causing a start codon (Methionine) to change to a stop codon in codon 605 of the transmembrane domain (**Figure 2B**). Svensk et al. revealed that *iglr-2* mutant exhibited a withered tail tip defect when grown at 15°C for 144 h (34). Consistent with their findings, we also observed that four of our *iglr-2* alleles shared the same withered tail tip defect phenotype at 15°C (**Figure S2**), implying the function of IGLR-2 is disrupted in these mutants. We next examined the susceptibility of these *iglr-2* null mutants to EHEC. *iglr-2* mutants disrupted either in LRR domain or transmembrane domain were more susceptible to EHEC compared to the wild-type N2 (**Figures 2C, D**, All *P* < 0.001 by Log-rank test). Moreover, the colonization of EHEC in *iglr-2(wf275)* was significantly increased (**Figure S3A**), but the defecation cycle and pumping rate were compatible to N2 (**Figures S3B, C**), which implied that the increased bacterial accumulation was not contributed by rapid pumping and/or slow defecation in *iglr-2(wf275)* animals. Taken together, our data indicated that *iglr-2* loss-of-function in *C. elegans* confers animals hypersusceptibility to EHEC infection.

### Overexpression of *iglr-2* Confers *C. elegans* Resistance to EHEC

**Figure 3:**
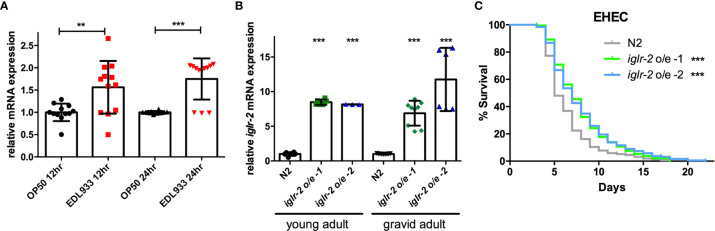
Overexpression of *iglr-2* confers *C. elegans* resistance to EHEC. **(A)** *iglr-2* transcriptional level is significantly up-regulated upon EHEC infection. The *iglr-2* mRNA expression level of N2 worms infected with *E. coli* O157:H7 strain EDL933 at 20°C was measured by qRT-PCR analysis at 12 h p.i (hours post infection) or 24 h p.i. Results were the average of three biological replicates and were normalized to the expression level of the *nhr-23* control gene. Expression is relative to *E. coli* OP50. The error bars represent the standard deviation. **P < 0.01 and ***P < 0.001 by the unpaired t-test, respectively. **(B)** The mRNA expression level of *iglr-2* in *iglr-2* overexpressed strains was measured by qRT-PCR analysis. cDNA was extracted from N2 and two overexpressed strains, *iglr-2* o/e-1 and *iglr-2* o/e-2, which were fed with OP50 and grown to young adult stage or gravid adult stage at 20°C. Results were normalized to the expression level of the *nhr-23* control gene. Expression is relative to wild-type N2. ***P < 0.001 by the unpaired t-test. **(C)** Survival curves of two *iglr-2* overexpressed strains feeding on EHEC exhibited a significant resistant phenotype compared to that of N2 (*iglr-2* o/e-1 *P* < 0.001, *iglr-2* o/e-2 *P* < 0.001) at 20°C. ***P < 0.001 by the Mantel–Cox log-rank test. Survival curves represent the sum of animals in multiple experiments.

To examine the relationship between *iglr-2* expression and EHEC infection, we detected the change in the expression level of *iglr-2* after EHEC infection by qRT-PCR analysis. The data demonstrated that *iglr-2* transcript expression level was significantly upregulated after wild-type N2 animals fed on EHEC for either 12 h post infection (h p.i.) (*P* < 0.01 by unpaired t-test) or 24 h p.i. (*P* < 0.001 by unpaired t-test) compared to that of the control OP50 (**Figure 3A**). These results indicated that *iglr-2* is induced upon EHEC infection. We were then interested in the effect of *iglr-2* overexpression in *C. elegans*. We constructed the *iglr-2* expression plasmid with whole *iglr-2* sequence and injected the plasmid into wild-type N2 to generate two independent *iglr-2* overexpressed strains *iglr-2* o/e-1 (strain YQ322) and *iglr-2* o/e-2 (strain YQ323). First, we examined whether the expression level of *iglr-2* was elevated in these overexpressed strains by qRT-PCR analysis. Expression level of *iglr-2* transcripts in *iglr-2* o/e-1 and *iglr-2* o/e-2 was significantly enhanced regardless of feeding on OP50 to young adult stage (both of *P* < 0.001 by unpaired t-test) or gravid adult stage (both of *P* < 0.001 by unpaired t-test) (**Figure 3B**). Next, we fed N2, *iglr-2* o/e-1 and *iglr-2* o/e-2 with EHEC to examine their sensitivity to EHEC. The results showed that *iglr-2* overexpressed strains were significantly more resistant to EHEC compared to that of N2 (**Figure 3C**, both *P* < 0.001 by Log-rank test). Taken together, our data suggested that *iglr-2* plays an important role in protecting *C. elegans* against EHEC infection.

### *iglr-2* Is Expressed in Neuronal and Intestinal Cells and May Act in These Tissues to Defend Against EHEC

**Figure 4:**
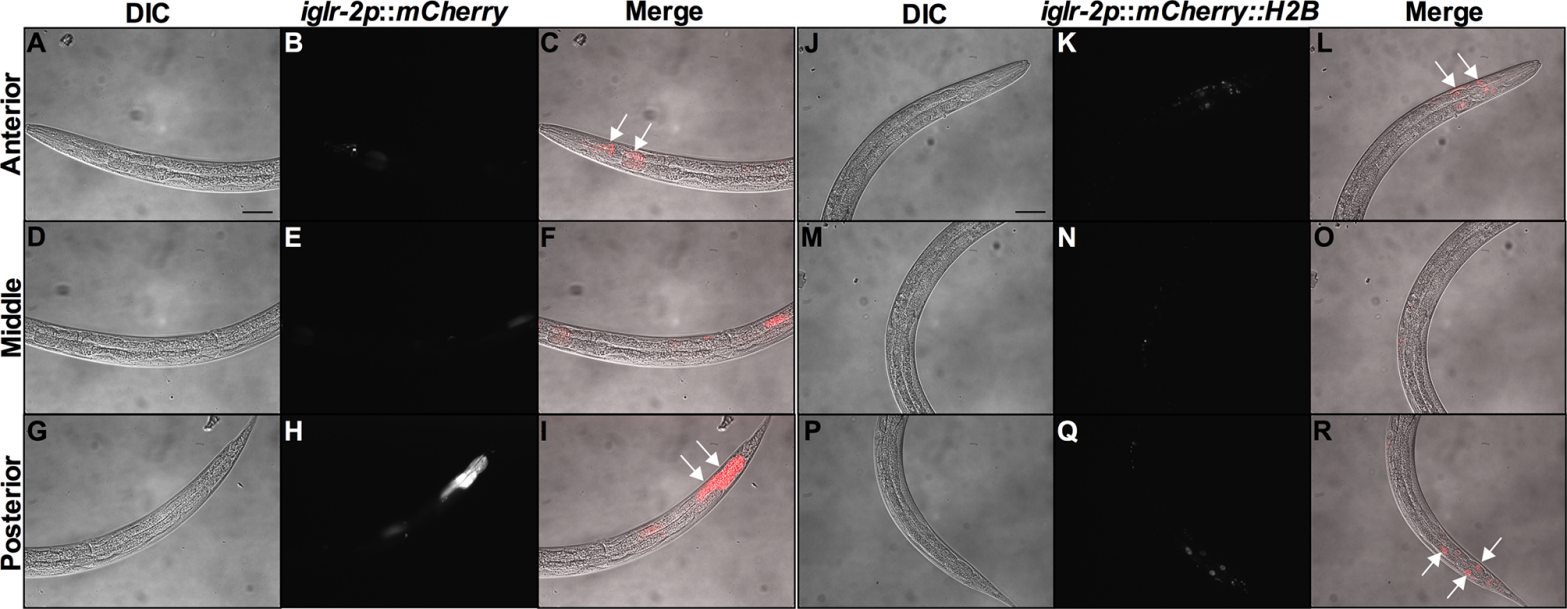
*iglr-2* is expressed in neurons and intestine. Fluorescence images show *iglr-2p*::*mCherry* or *iglr-2p*::*mCherry*::H2B reporter strains exhibiting mCherry fluorescent signal in both neuronal and anterior/posterior intestinal cells at the young adult stage. **(A–I)** Representative images of YQ305 [*iglr-2p::mCherry*] transgenic animals are shown. **(A–C)** Anterior part of *C. elegans* YQ305 strain is shown. Arrows indicate the neuronal cells of head and anterior part of intestinal cells in **(C)**. **(D–F)** Middle part of *C. elegans* YQ305. **(G–I)** Posterior part of *C. elegans* YQ305 strain is shown. Arrows indicate the posterior part of intestinal cells in **(I)**. **(A, D, G)** represent differential interference contrast (DIC) images of the proximal, middle, and distal parts of *C. elegans*, respectively. **(B, E, H)** mCherry fluorescence images of *iglr-2p*::*mCherry* animals. **(C, F, I)** Merged images of *iglr-2p*::*mCherry* animals. **(J–R)** Representative images of YQ326 [*iglr-2p::mCherry::H2B*] transgenic animals are shown. **(J–L)** Anterior part of *C. elegans* YQ326 strain is shown. Arrows indicate the neuronal nuclei of head and anterior part of intestinal nuclei in **(L)**. **(M–O)** Middle part of *C. elegans* YQ326. **(P–R)** Posterior part of *C. elegans* YQ326 strain is shown. Arrows indicate the posterior part of intestinal nuclei in **(R)**. **(J, M, P)** represent differential interference contrast (DIC) images of the proximal, middle, and distal parts of *C. elegans*, respectively. **(K, N, Q)** mCherry fluorescence images of *iglr-2p*::*mCherry* animals. **(L, O, R)** Merged images of *iglr-2p*::*mCherry* animals. All the scale bars represent 50 µm.

**Figure 5:**
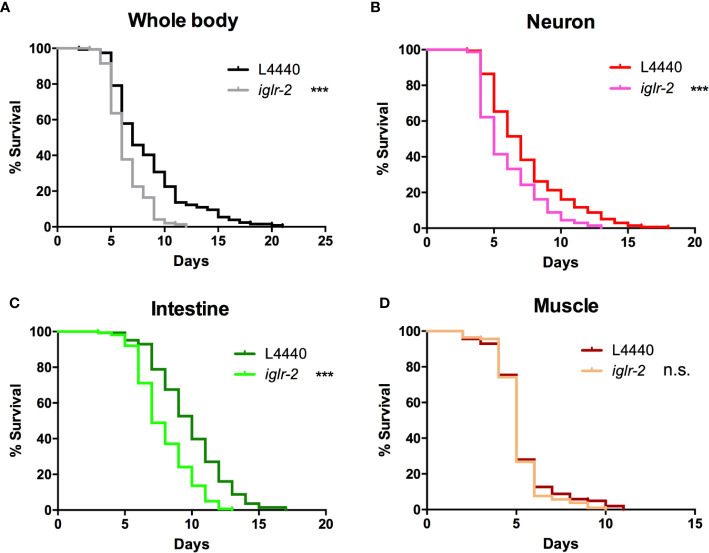
*iglr-2* may act in the neuron and intestine to defend against EHEC. **(A)** Survival curve of RNAi-sensitized strain NL2099 *rrf-3*(pk1426) showed that *iglr-2* silencing in whole-body cell resulted in *C. elegans *being more sensitive to EHEC infection compared to that of empty vector L4440 control (*P* < 0.001). **(B)** Survival curve of neuron-specific RNA knockdown strains TU3401 showed that *iglr-2* silencing in neuron cells resulted in *C. elegans* is more sensitive to EHEC compared to that of empty vector L4440 (*P* < 0.001). **(C)** Survival curve of intestine-specific RNAi knockdown strain VP303 showed *iglr-2* silencing in intestine cells resulted in *C. elegans* being more sensitive to EHEC infection compared to that of empty vector L4440 control (*P* < 0.001). **(D)** Specific knockdown of *iglr-2* in muscle cells by muscle-specific RNAi knockdown strain WM118 showed a similar survival curve compared to that of empty vector L4440 control to EHEC infection (*P* = 0.3725). ***P < 0.001 by the Mantel**–**Cox log-rank test. Survival curves represent the sum of animals in multiple experiments.

In order to determine the expression pattern and identify the site of action of *iglr-2*, we constructed a plasmid that specifically expresses red fluorescence under the control of the *iglr-2* 5′ promoter and injected the plasmid into wild-type animals to generate two independent transcriptional reporter animals *iglr-2p::mCherry* (strain YQ305) and *iglr-2p::mCherry::histone H2B* (strain YQ326). We fed these two transcriptional reporter strains with *E. coli* OP50 and examined the expression pattern of red fluorescence at their young adult stage. *iglr-2p::mCherry* transgenic animals displayed red fluorescence expression in the neurons of the head and anterior/posterior part of the intestinal cells, which suggested that *iglr-2* is expressed in these tissues (**Figures 4A–I**). *iglr-2p::mCherry::histone H2B* transgenic animals also displayed red fluorescence expression pattern resembling *iglr-2p::mCherry* animals except for the red fluorescent signal only being detected in the nuclei of these tissues (**Figures 4J–R**). Furthermore, *iglr-2p::iglr-2a::mCherry* (strain YQ338) and *iglr-2p::iglr-2b::mCherry* (strain YQ362) animals showed two IGLR-2 protein isoforms, which were also expressed in the neurons of the head and anterior/posterior part of the intestine (**Figure S4**). To reconfirm the site of action of *iglr-2*, we used tissue-specific RNA knockdown strains to silence *iglr-2* in specific tissues and then examined their survival on EHEC infection. **Figure 5A** shows the survival curves of *iglr-2* silencing in RNAi-sensitized strain *rrf-3(pk1426)* resulting in *C. elegans* exhibiting susceptible phenotype to EHEC compared to that of empty vector control, L4440 (*P* < 0.001 by Log-rank test). Similar results are shown in **Figures 5B, C**; when knockdown of *iglr-2* was specific to neurons or the intestine in worms, they were significantly more susceptible to EHEC (*P* < 0.001 by Log-rank test). However, specific knockdown of *iglr-2* in the muscle did not alter the susceptibility of animals to EHEC compared to the empty vector control (**Figure 5D**, *P* = 0.3725 by Log-rank test). These data are consistent with the images (**Figure 4**) that show that *iglr-2* is only expressed in the neurons and the intestines. Taken together, these data suggested that *iglr-2* plays a role in the neural system and intestine to defend against EHEC.

### *iglr-2* Is Required for Behavioral Avoidance of EHEC

**Figure 6:**
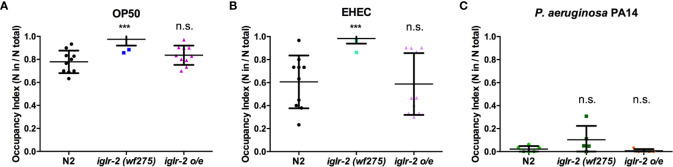
*iglr-2* is required for bacterial avoidance behavior to EHEC. **(A)** Occupancy index of wild-type N2, *iglr-2* mutants, and *iglr-2* overexpressed strains on *E. coli* OP50 bacterial lawn for 16 h was analyzed. *iglr-2* mutant strain lost the ability to avoid OP50 compared to that of wild-type N2 by unpaired t-test (***, N2 vs. *iglr-2 (wf275), P* < 0.001, N2 *vs* *iglr-2* o/e-1 *P* = 0.173). **(B)** Occupancy index of wild-type N2, *iglr-2* mutants and *iglr-2* overexpressed strains on EHEC bacterial lawn for 16 h was scored. *iglr-2* mutants displayed higher occupancy on EHEC compared to that of wild-type N2 by unpaired t-test. *** indicates N2 *vs*. *iglr-2 (wf275) P* < 0.001 and ns (no significance) indicates N2 *vs* *iglr-2* o/e-1 *P* = 0.871. **(C)** Occupancy index of wild-type N2, *iglr-2* mutants, and *iglr-2* overexpressed strains on *P. aeruginosa* PA14 bacterial lawn for 16 h was scored. *iglr-2* mutants and overexpressed strain showed a similar avoidance behavior index compared to that of wild-type N2 by unpaired t-test [*iglr-2 (wf275)* *P* = 0.140, *iglr-2* o/e-1 *P* = 0.289]. ns indicates no significance. Each experiment conducted on three biological replicates, and results were the average of experimental replicates. Error bars represent SD.

We noticed that part of the *iglr-2* signal appears in the neurons (**Figure 4** and **Figure S4**). In the *C. elegans* nervous system, the olfactory nerve has been reported to play an important role in detecting pathogens and to mediate host–pathogen interactions through chemosensation (29). *C. elegans* is attracted by most bacteria, but some pathogens like *P. aeruginosa* stimulate *C. elegans* to avoid them (35). We demonstrated that the *iglr-2* expression was located with neurons in the head and tail (**Figure 4** and **Figure S4**). Therefore, we hypothesize that *iglr-2* may be involved in avoidance behavior to EHEC. We transferred N2, *iglr-2* mutant and *iglr-2* overexpressed worms to the plates containing bacterial lawn and scored the occupancy of animals on the bacterial lawn after keeping them at 20°C for 16 h. The occupancy index was calculated as the number of animals on the bacterial lawn divided by the total number of animals on plates. Indeed, loss of *iglr-2* resulted in more animals lingering on OP50 (*P* < 0.001 by unpaired t-test) or EHEC (*P* < 0.001 by unpaired t-test) bacterial lawn compared to that of wild-type N2 animals (**Figures 6A, B**). Overexpression of *iglr-2* reversed the avoidance behavior, which was similar to wild-type N2 on either the OP50 or EHEC bacterial lawn (**Figures 6A, B**). Of note, *iglr-2* deficiency animals exhibited similar avoidance behavior to *P. aeruginosa* PA14 compared to that of wild-type N2 (**Figure 6C**, P = 0.14 by unpaired t-test), which suggested that *iglr-2* showed selectivity to *E. coli* and was not involved in the avoidance behavior to *P. aeruginosa* in *C. elegans*. Taking these results together, we demonstrated that *iglr-2* is required for behavioral avoidance to pathogenic *E. coli*, EHEC, and the relatively weak pathogen, *E. coli* OP50 (36) in *C. elegans*. *P. aeruginosa* PA14 is considered an acute pathogen to *C. elegans* which kills animals within one day (37). We noticed that animals on PA14 bacterial lawn showed a low percentage (~0%) of occupancy for exposing 16 h. To test whether the fast killing of PA14 may hinder the potential increased avoidance of animals, we reduced the assay time of avoidance behavior experiment. We first scored the occupancy index of N2 animals on PA14 in a time-dependent manner. The occupancy of N2 on PA14 was approximately 40% for 8 h exposure and was dropped to 10% for 12 h (**Figure S5**). We then measured the occupancy of *iglr-2* mutant on PA14 for 8-h treatment and found that *iglr-2(wf275)* exhibited a comparable aversion behavior compared to N2 (**Figure S6**, P = 0.15 by unpaired t-test), suggesting that the occupancy assay might not be affected by fast killing of PA14.

### p38 MAPK Pathway Acts Downstream of *iglr-2*

**Figure 7:**
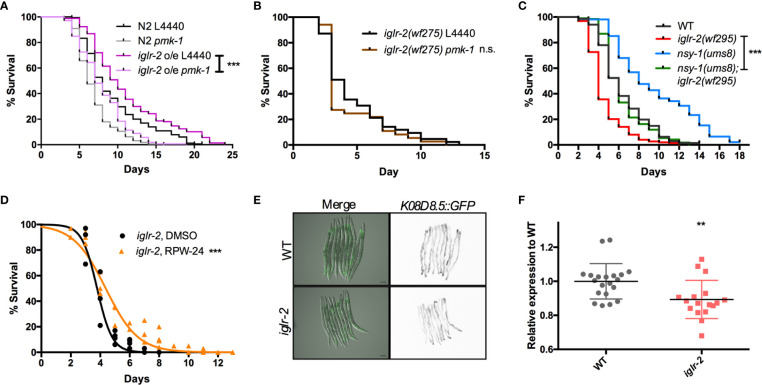
PMK-1 p38 mitogen-activated protein kinase (MAPK) pathway acts as downstream signal of *iglr-2*. **(A)** RNAi-mediated knockdown of *pmk-1* in *iglr-2* overexpressed animal resulted in hypersusceptibility to EHEC compared to empty vector control, L4440 (*P* < 0.001). ***P < 0.001 by the Mantel**–**Cox log-rank test. **(B)** Survival curves of *iglr-2* mutant RNAi against *pmk-1* were comparable to EHEC compared to empty vector control, L4440 (*P* = 0.22) **(C)** Survival of *nsy-1(ums8)* gain-of-function allele and *iglr-2(wf295)* double mutant strain infected with EHEC at 20°C was examined. *nsy-1(ums8);iglr-2(wf295)* animals lived longer than *iglr-2(wf295)* (*P* < 0.0001) when fed with EHEC at 20°C but exhibited a similar survival compared to that of wild-type (WT) worms (*P* = 0.835). *nsy-1(ums8)* gain-of-function strain was significantly resistant to EHEC infection compared to that of WT (*P* < 0.001). ***P < 0.001 by the Mantel**–**Cox log-rank test. **(D)** Survival analysis of *iglr-2(wf295)* supplemented with 5 µM RPW-24 upon EHEC infection at 20°C was examined. Treatment of RPW-24 increased the survival of *iglr-2(wf295)* animals compared to that of DMSO control. (*P* < 0.001). Survival curves represent the sum of animals in multiple experiments. **(E)** Representative images of *K08D8.5::GFP* transgenic animals infected with EHEC were shown. *K08D8.5::GFP* wild type and *iglr-2* mutant background worms were treated with EHEC for 8 h and imaged. Merged images indicate GFP overlaid with DIC. GFP images are presented inverted signal of GFP. Scale bars represent 100 µm. **(F)** Relative fluorescence expression of *K08D8.5::GFP* transgenic worms infected with EHEC for 8 h. Each dot represents the relative GFP expression of single animal to the mean of wild-type control. **P < 0.01 by the unpaired t-test. Error bars represent SD.

Next, we aimed to identify the downstream signal of *iglr-2*. TOL-1, MOM-4, and PMK-3 function together in a signaling pathway in the chemosensory BAG neurons (29). Therefore, we were interested in whether the TLR signaling pathway is also involved in *iglr-2* protecting hosts from death by EHEC in *C. elegans.* We performed RNAi-mediated knockdown of *mom-4* in *iglr-2* overexpressed animals (*iglr-2* o/e, YQ322) upon infecting EHEC. Survival of *iglr-2* o/e with RNAi silencing of *mom-4*, a MAP kinase kinase kinase (MAP3Ks), in TLR signaling, was comparable to that of the empty vector control L4440 (**Figure S7**, P = 0.44 by Log-rank test), which suggested that the MAP3K- involved TLR signals are not the downstream signaling pathway of *iglr-2* in *C. elegans*. Several evolutionarily conserved innate immune responses have been reported to be involved in defending pathogenic microbes in *C. elegans*, for example, the insulin-like signaling pathway (38), the unfolded protein response (UPR) signaling pathway (38), the autophagy pathway (39), and the p38 MAPK pathway (38). We therefore tested these known innate immune pathways in *C. elegans* to determine whether these pathways are involved in the *iglr-2-*mediated defense system against EHEC. When insulin-like signaling pathway DAF-16/FOXO transcriptional factor, *daf-16*, was knocked down by RNAi-mediated silencing in *iglr-2* o/e, no significant difference was observed in the survival compared to empty vector, L4440 control (**Figure S8A**, P= 0.14 by Log-rank test). Similar results were also found when *xbp-1* was knocked down in *iglr-2* o/e, which is spliced by IRE-1 during endoplasmic reticulum (ER) stress to activate UPR. The survival was compatible to the L4440 control (**Figure S8B** *, P* = 0.24 by Log-rank test). Knockdown of a helix–loop–helix transcription factor regulating autophagy gene expression, *TFEB*/*hlh-30*, in *iglr-2* o/e *C. elegans* resulted in slightly enhanced sensitivity compared to the control (**Figure S8C** *, P* < 0.05 by Log-rank test). Our previous study demonstrated that the p38 MAPK pathway is activated in *C. elegans* for defending EHEC infection (19). To determine whether the p38 MAPK pathway is involved in *iglr-2* regulating immune response, we examined the survival of *iglr-2* o/e strain silencing of genes involved in p38 MAPK pathway upon EHEC infection. *iglr-2* o/e subjected to *pmk-1* RNAi was significantly susceptible to EHEC compared to the empty vector control, L4440 (**Figure 7A**, *P* < 0.001 by Log-rank test). Consistently, RNAi knockdown of *nsy-1*, *tir-1* and *sek-1* suppressed the extended survival of *iglr-2* o/e to EHEC, respectively (**Figures S9A–C**). Furthermore, survival of RNAi against *pmk-1* to *iglr-2(wf275)* was compatible to that of control (**Figure 7B**, *P* = 0.22 by Log-rank test), suggesting *iglr-2* and *pmk-1* act in the same pathway to defend EHEC in *C. elegans*. Together, these data indicated that the p38 MAPK signaling cascades act downstream of *iglr-2*. To further reconfirm the notion that the p38 MAPK signaling cascades act downstream of *iglr-2*, we performed genetic epistasis analysis using a *nsy-1* gain-of-function allele, *nsy-1(ums8)*, which constitutively activates the p38 MAPK cascades (31). We generated *nsy-1(ums8);iglr-2(wf295)* double mutant and examined its survival to EHEC. As shown in **Figure 7C**, hyperactivation of p38 MAPK pathway in *iglr-2(wf295)* significantly increased the survival of *iglr-2* mutant animals to EHEC infection. Moreover, *nsy-1(ums8);iglr-2(wf295)* animals were more susceptible to EHEC infection compared to *nsy-1(ums8)* (*P* < 0.001 by Log-rank test), and the survival curve of *nsy-1(ums8);iglr-2(wf295)* was similar to that of wild-type (WT) animals (*P* = 0.835 by Log-rank test). These results reconfirm that the *iglr-2*, at least partly, regulated the p38 MAPK pathway. An alternative explanation of this result is that p38 MAPK pathway hyperactivation also induces aversion behavior of *C. elegans* to EHEC, and disruption of *iglr-2* suppresses the behavior contributing *nsy-1(ums8);iglr-2(wf295)* susceptible to EHEC. However, we found that *nsy-1(ums8)* did not escape from EHEC infection, and *iglr-2* loss of function in *nsy-1(ums8)* animal exhibited a compatible aversion behavior to *iglr-2(wf295)* and *nsy-1(ums8)* (**Figure S10**), suggesting that suppression of the increased EHEC resistance in *nsy-1(ums8);iglr-2(wf295)* was not due to the absence of aversion behavior from *iglr-2* loss of function. In addition to activate the p38 MAPK immune pathway in the *iglr-2* mutant genetically, we also induced the p38 MAPK pathway pharmacologically to strengthen our hypothesis. RPW-24, a small molecule drug, had been reported to stimulate immune genes through p38 MAPK pathway in *C. elegans* (33). We therefore supplemented RPW-24 to *iglr-2* mutant worms upon EHEC infection and examined the survival. Treatment of 5 µM of RPW-24 increased the survival of *iglr-2* mutant animals against EHEC compared to the vehicle control (**Figure 7D**, *P* < 0.001). An interpretation of this result is that RPW-24 may directly inhibit or kill EHEC bacteria. However, EHEC treated with 5 µM RPW-24 exhibited similar growth curves to untreated control (**Figure S11**) suggesting that treatment of RPW-24 did not affect the growth of EHEC. Next, we asked whether the expression of p38 MAP kinase-dependent genes is changed in *iglr-2* mutant. We examined the GFP expression of *K08D8.5::GFP* strain, a gene that encoded CUB-like domain regulated by the p38 MAPK signaling (32), under *iglr-2(wf275)* background. The expression pattern of *K08D8.5::GFP* was throughout the intestine of the worms extensively while subjected to EHEC (**Figure 7E**). In contrast, *iglr-2(wf275)*;*K08D8.5::GFP* strain showed a significant reduction of GFP level to wild-type control (**Figures 7E, F**, *P* < 0.01). These results indicated that *iglr-2* is involved in the regulation of p38 MAPK-dependent gene. It did not escape our attention that *iglr-2* had been reported to regulate NHR-49 (34) (ortholog of the mammalian nuclear-hormone receptor PPAR*α*), which is correlated with *C. elegans’* immune response (40, 41). We then examined the susceptibility of *iglr-2* o/e *C. elegans* RNAi knockeddown of *nhr-49* to EHEC infection. *iglr-2* o/e treated with *nhr-49* RNAi lived significantly shorter than the control (**Figure S12**, *P* < 0.001), suggesting that *nhr-49* might be one of the downstream of *iglr-2* in *C. elegans* defending EHEC. All together, our results demonstrated that *iglr-2* is required for the host defense in *Caenorhabditis elegans*, at least in part, through the p38 MAPK immune pathway.

## Discussion and Conclusions

**Figure 8:**
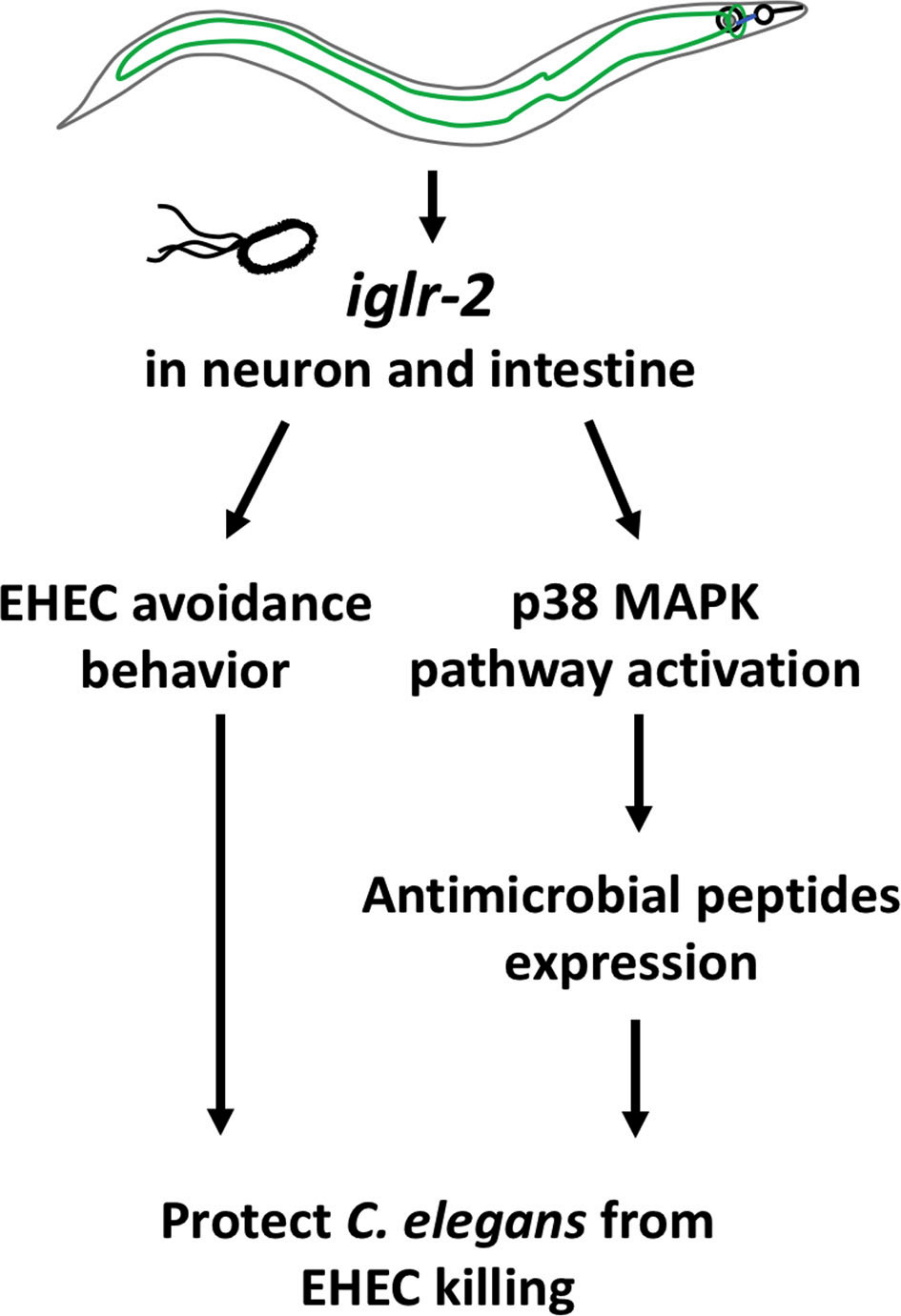
Model graphic of *iglr-2* defense against EHEC in *C. elegans*. IGLR-2, an transmembrane protein containing immunoglobulin-like and leucine rich repeat domains, is involved in *C. elegans’* avoidance behavior to EHEC and triggering downstream p38 MAPK pathway which might function in neuronal cell and intestinal cell.

In this study, we identified that a membrane protein, IGLR-2, containing a leucine-rich repeat (LRR) domain, is required for host defense in *C. elegans*. Our genetic analysis demonstrated that loss of *iglr-2* conferred susceptibility of *C. elegans* to EHEC infection, and overexpression of *iglr-2* contributed to animal host resistance to EHEC. *iglr-2* was also required for avoidance behavior to EHEC infection. Moreover, we demonstrated that *iglr-2* might act in neural and intestinal tissues and be involved in regulating p38 MAPK pathway activation, partly as a downstream immune response to protect *C. elegans* from EHEC death (**Figure 8**). IGLR-2 has been reported to be a plasma membrane sensor interacting with PAQR-2 protein regulating the membrane fluidity by promoting fatty acid desaturation to respond to decreased fluidity during cold adaptation or a diet rich in saturated fatty acids (34, 42, 43). Here, we report our knowledge for the first time that *iglr-2* takes part in response to pathogen infection.

In our PRR RNAi library screening, we identified seven potential genes (*mog-2, soc-2*, *pxn-2*, *sds-22*, *lron-9, zmp-2*, and *iglr-2*) involved in *C. elegans* against EHEC. *mog-2* encodes a U2 snRNP protein and is involved in sex determination, meiosis, and splice site selection in *C. elegans* (44). *soc-2* encodes a leucine-rich repeat protein, which is involved in the fibroblast growth factor receptor signaling pathway and is positively regulated by *let-60/Ras* and negatively regulated by *egl-15/FGF* (FGF receptor) in development (45, 46). *pxn-2* encodes peroxidasin (a family of extracellular peroxidases) which is essential for embryonic morphogenesis in *C. elegans* (47). *sds-22* is a phosphatase regulator gene, and loss of *sds-22* causes embryonic lethality in *C. elegans* (48, 49)*. lron-9* is predicted as a glycoprotein that contains an extracellular leucine-repeat and transmembrane region, which is expressed at the head neuron, muscles, and seam cells, and knockdown of *lron-9* by RNAi leads to developmental defect in worms (50, 51). ZMP-2 is a zinc metalloproteinase which is responsible for development and pathogen resistance in *C. elegans* (52). Of note, *zmp-2* was reported having an immune related function, which strengthens the reliability of our PRR RNAi library screening. Nevertheless, further research of the other five genes (*mog-2, soc-2, pxn-2, sds-22* and *lron-9*) in immune regulation is warranted.

We noticed that the *iglr-2* expression pattern of our transgenic animals is distinct from the previous research (34). One plausible interpretation is that the promoter region used here is different from the previous one. The *mrps-18A* gene is adjacent to the promoter of *iglr-2*. Svensk et al. used the region of ~4 kb upstream of *iglr-2* as an *iglr-2* promoter also includes the upstream operon gene, *mrps-18A* (34). To avoid the effect of *mrps-18A*, we only cloned the 218 bp fragment from the end of the 3′ untranslated region (UTR) of *mrps-18A* to the start site of *iglr-2* as the *iglr-2* 5′UTR/promoter for our *iglr-2* transgene. Another potential explanation is the operation of different 3′UTR, in which we chose *unc-54* for our construct, even though the use of *unc-54* 3′UTR for a transgene is concerned with an artificial expression signal in the posterior of the intestine in *C. elegans* (53). Nevertheless, we not only observed the mCherry signals in the posterior intestine but also in the anterior intestine and neurons. These results suggested that the mCherry signals are less likely to be the artifacts as the consequence of using the *unc-54* 3′ UTR.

*C. elegans* animals with *iglr-2* mutation occupied the EHEC bacterial lawn longer than N2 but showed similar occupancy to N2 on *P. aeruginosa* bacterial lawn (**Figure 6**). These data indicate that mutation of *iglr-2* does not affect the function of neuron or muscle to abolish the movement in *C. elegans*, but that *iglr-2* gene is specific for triggering *C. elegans* host avoidance behavior to minimize EHEC killing. In other words, *C. elegans* is able to distinguish different pathogens, initiating a unique response to protect itself from pathogen attack. TOL-1 is the sole toll-like receptor homolog in *C. elegans.* Currently, TOL-1 does not appear to participate in regulating canonical immune response but plays a role in behavioral avoidance of pathogens (29). *tol-1* mutants are defective in the terminal differentiation and function of the BAG neurons which are necessary for chemosensitivity to CO_2_, resulting in their pathogen-avoidance behavior (54). Although *iglr-2* is also involved in pathogen-avoidance behavior, no evidence suggests that *iglr-2* is involved in neuron differentiation and function; however, whether *iglr-2* mutants are defective in sensing CO_2_ warrants examination. Furthermore, *tol-1* seems not to trigger the innate immune pathway according to current studies (54, 55). Based on our epistasis results (**Figure 7**), we found that p38 MAPK pathway, at least in part, is involved in the downstream of *iglr-2* against EHEC infection, implying that *iglr-2* might be a potential PRR in *C. elegans*.

A recent study showed that a novel protein, CgLRRIG-3, which contains leucine-rich repeat (LRR) and immunoglobulin-like (Ig) domains, plays a crucial role in regulating immune response in oyster, *Crassostrea gigas* (56). CgLRRIG-3 can bind to lipopolysaccharide (LPS), peptidoglycan (PGN), glucan (GLU), and polyinosinic:polycytidylic acid (poly I:C) to stimulate cytokine expression and enhance the phagocytosis rate of the hemocytes in *Crassostrea gigas.* This research revealed that the proteins containing leucine-rich repeat (LRR) and immunoglobulin-like (Ig) domains have unidentified functions in immune response in invertebrates. Taken together our findings expand understanding of how *C. elegan*s mounts immune response upon facing pathogens and also advance our current knowledge about evolutionarily conserved strategies in invertebrates.

## Data Availability Statement

The original contributions presented in the study are included in the article/**Supplementary Material**. Further inquiries can be directed to the corresponding authors.

## Ethics Statement

Ethical review and approval was not required for the animal study because the authors used *C. elegans*, the nematode as an animal model, which is not required ethical review and approval for study.

## Author Contributions

Conceived and designed the experiments: C-JK and C-SC. Performed the experiments: C-JK, B-YL, Y-CH, SL, and S-TW. Analyzed the data: C-JK, B-YL, Y-CH, SL, Y-WC, S-TW, and C-SC. Contributed reagents/materials/analysis tools: C-JK, B-YL, Y-CH, SL, and S-TW. Wrote the paper: C-JK and C-SC. All authors contributed to the article and approved the submitted version.

## Funding

This work is supported by the Ministry of Science and Technology (MOST) grants (106-2321-B-006-005- and 107-2628-B-006-003-) to C-SC. The funders had no role in the study design, data collection, and analysis, decision to publish, or preparation of the manuscript.

## Conflict of Interest

The authors declare that the research was conducted in the absence of any commercial or financial relationships that could be construed as a potential conflict of interest.
