# Supplementary material for: IGLR-2, a Leucine-Rich Repeat Domain Containing Protein, Is Required for the Host Defense in Caenorhabditis elegans
Source: Front Immunol. 2020 Nov 30;11:561337. doi: 10.3389/fimmu.2020.561337 (PMC7734252; doi:10.3389/fimmu.2020.561337)
Supplement: Supplementary file 1 [file DataSheet_1.pdf]

Supporting information

Experimental Procedures:

Screening of the PRRs RNAi library

PRRs RNAi library, stored in 96-well plates and in -80°C freezers, was replicated in LB broth containing 50 μg/mL carbenicillin (Carb) with a 96-well pin replicator and cultured in a 37°C incubator for 16 to 18 hours at Day 1. At Day 2, the entire library was triplicated in 96-well plates containing LB broth with 50 μg/mL Carb and cultured at 37°C for another 16 to 18 hours. At the same day, the eggs of *C. elegans rrf-3(pk1426);glp-4(bn2)* mutant were collected by the standard alkaline hypochlorite method. At Day 3, 1 mM of IPTG was added to each well of 96-well plates for inducing double-stranded RNA and put in a 37°C incubator. One hour later, RNAi bacteria were centrifuged and resuspend in S medium (S medium: 5.85 g/L NaCl, 6 g/L KH_2PO_4, 1 g/L K_2HPO_4, 30mM $MgSO_4$, 30 mM $CaCl_2$, 5 ng/L cholesterol, 10 mM potassium citrate, 50 nM EDTA, 25 nM $FeSO_4$, 10 nM $MnCl_2$, 10 nM $ZnSO_4$, 1 nM $CuSO_4$) and were transferred from 96-well format to 48-well plates. Approximately 20 synchronized *rrf-3(pk1426); glp-4(bn2)* larval 1 stage (L1) worms were placed in each well and allowed to develop to L4 stage at 25°C with gentle shaking. The *rrf-3(pk1426); glp-4(bn2)* mutant nematodes are RNAi-sensitive (contributed by *rrf-3*) and are defective in germ-line proliferation (contributed by *glp-4*) at the restrictive temperature (25 °C). At Day 5, when *C. elegans rrf-3(pk1426);glp-4(bn2)* animals reached to L4 larvae/young adult stage, overnight cultured *E. coli* OP50 or EDL933 bacteria were resuspended in S medium containing 1 mM IPTG and mixed to RNAi-fed worms in each well. The plates were covered with porous Rayon films to allow air exchange and placed back at 25°C with shaking gently. After 12 days, the survival of animals was scored by visualization under dissecting microscopes. For each batch of RNAi clones tested, empty vector (L4440), *daf-2* and *sek-1* RNAi clones were included as negative (no knockdown) and positive (resistant, susceptible) controls respectively. The survival rate of OP50/L4440 group (as a health monitoring control) was approximately 83%; EDL933/L4440 group was approximately 52%; EDL933/*daf-2* group was approximately 93% and EDL933/*sek-1* group was approximately 17%. The RNAi hits/candidates with an increased mortality toward *C. elegans* were selected with survival rate that was significantly lower compared to the EDL933/L4440 group ($P < 0.05$).

Withered tail tip images

For imaging withered tail of *iglr-2* mutants, synchronized L1 worms were placed on plates and incubated at 15 °C for 6 days as described (Svensk et al., 2013). Animals were mounted on glass slides with 2 % agarose pads and anesthetized with 25 nM sodium azide (NaN3). The withered tail tip phenotype was observed by differential

interference contrast (DIC) imaging with Nomarski optics using a Nikon Eclipse Ti inverted microscope system with DP72 CCD camera.

Pharyngeal pumping rate
Measurement of pharyngeal pumping rate in *C. elegans* was performed as described with slight modifications (Kroetz et al., 2012). In brief, the pharyngeal pumping rate of *C. elegans* feeding on OP50 was measured by counting the contractions of the terminal pharyngeal bulb of individual *C. elegans* animal. Each worm was measured for 10 seconds using an Olympus stereo microscope at 90x magnification**.** The pharyngeal pumping was defined as described (Avery and Shtonda, 2003).

Defecation
Measurement of defecation cycle length in *C. elegans* was performed as described with slight modifications (Branicky et al., 2001). Briefly, the defecation cycle length was scored in the L4 hermaphrodites incubated on OP50 at 20°C. Each animal was measured for 10 minutes under an Olympus stereo microscope at 90x magnification and the defecation cycle was defined as described (Branicky et al., 2001).

Quantification of bacterial intestinal colonization
Measurement of bacterial number colonized in the intestine of *C. elegans* was performed as our previous published procedures (Chou et al., 2013; Kuo et al., 2016; Kuo et al., 2018). In brief, animals fed with either GFP-labeled *E. coli* OP50 or *E. coli* O157:H7 EDL933 at 20 °C for one day and then transferred to the OP50 bacterial plates for another two days at 20°C. The infected animals were washed out from the plates, treated with 25 mM levamisole and washed in M9 medium 10 times. The infected animals were then treated with M9 buffer containing 25 mM levamisole, 100 μg/ml gentamicin, and 0.5 mg/mL carbenicillin for 1 to 2 hours at room temperature. These antibiotics were eliminated by washing the worms in M9 buffer with 25 mM levamisole 3 times. After the final wash, ten worms were picked randomly into 100 μL M9 buffer in an eppendorf microtube, pulverized for 1 minute using a sterile plastic pestle, and plated on LB agar containing ampicillin after serial dilution. The number of bacterial cells (colony number) was determined and the colony-forming unit (CFU) per worm was calculated.

Figures

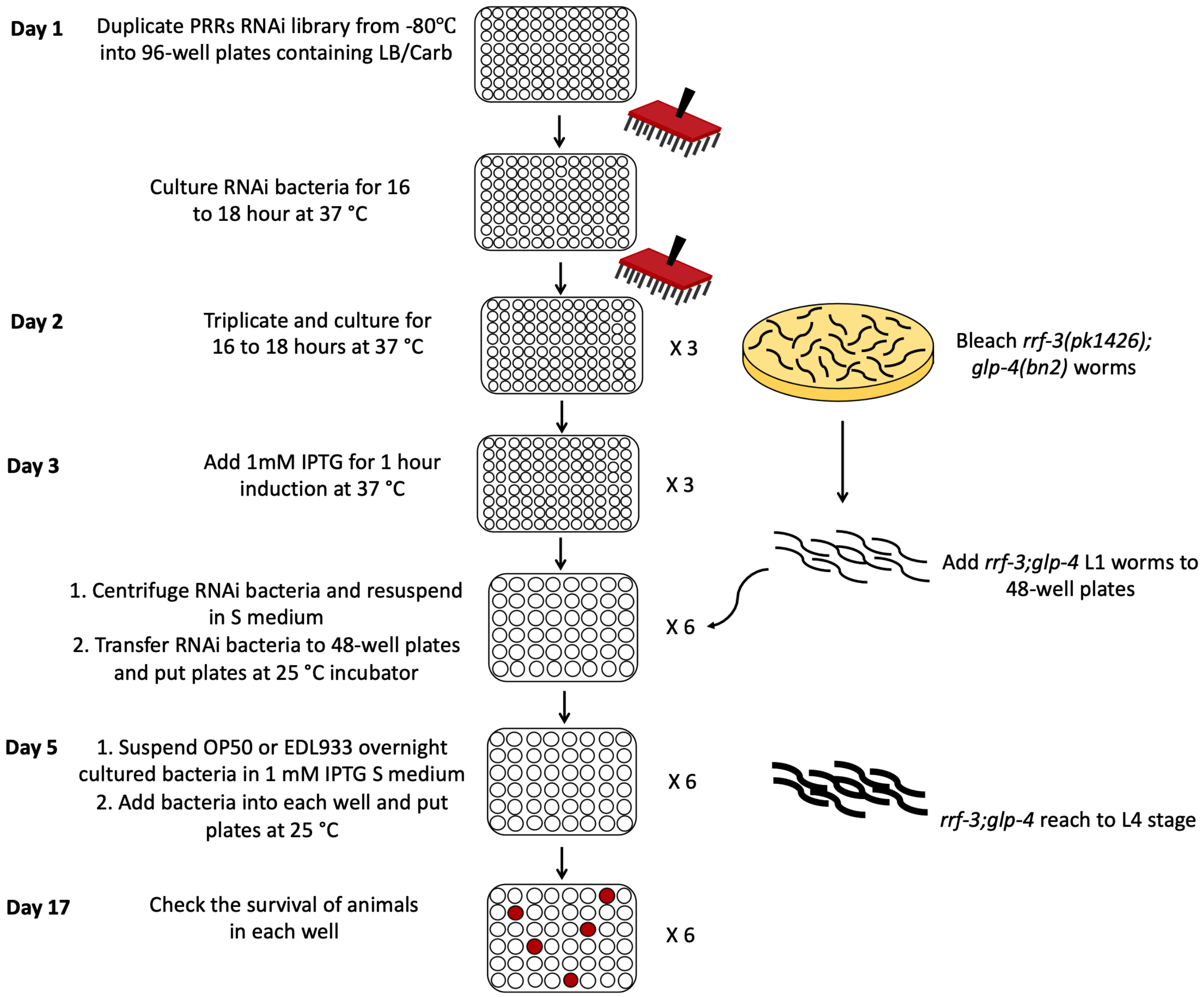

Figure S1. Flow chart of RNAi screening. Detail procedure is described in the Experimental Procedures.

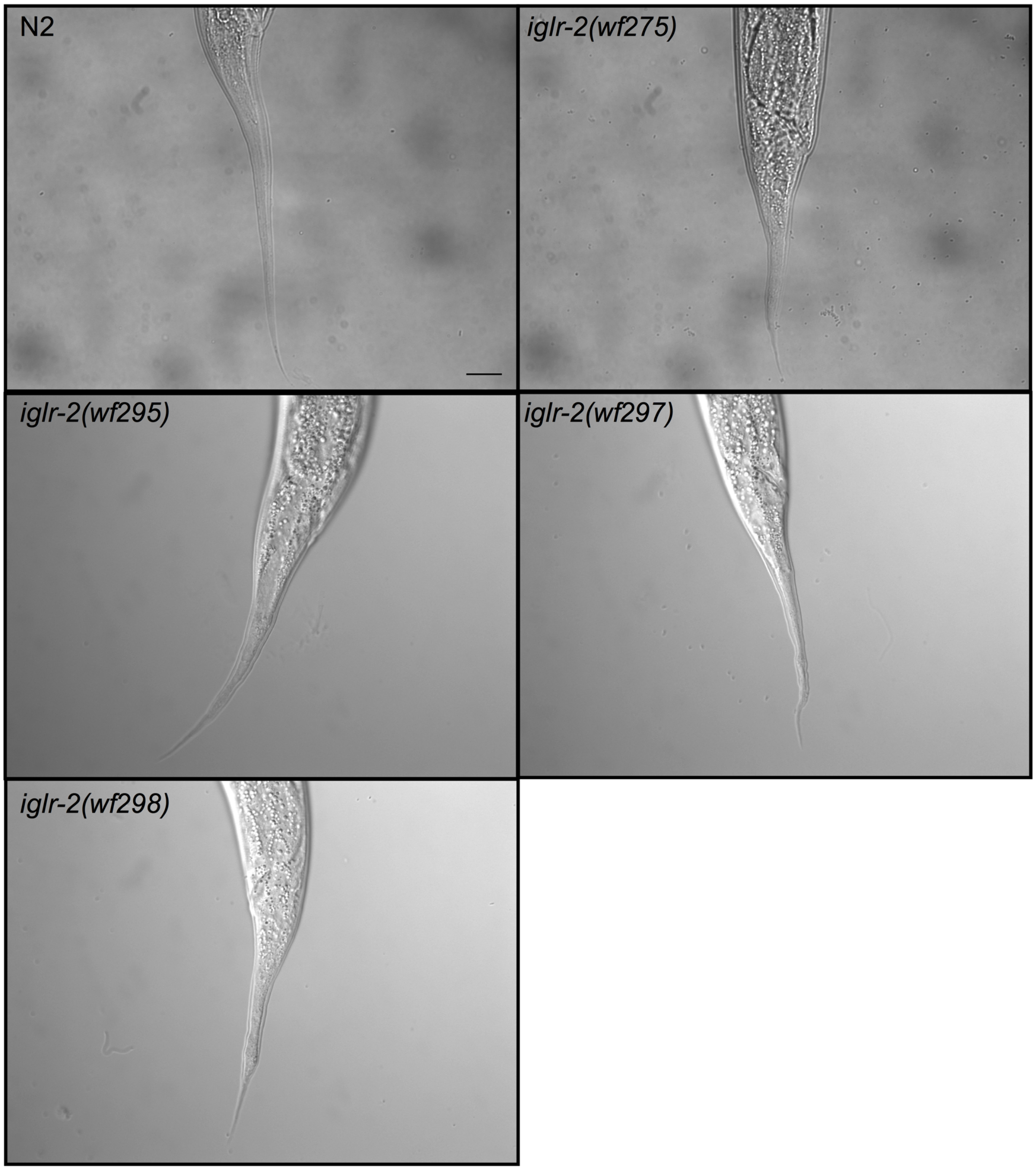

Figure S2. *iglr-2* mutant alleles exhibit a withered tail tip defect at 15 °C. The images of withered tail of four *iglr-2* mutants incubated at 15°C for 6 days are shown. Scale bar represents 20 μm.

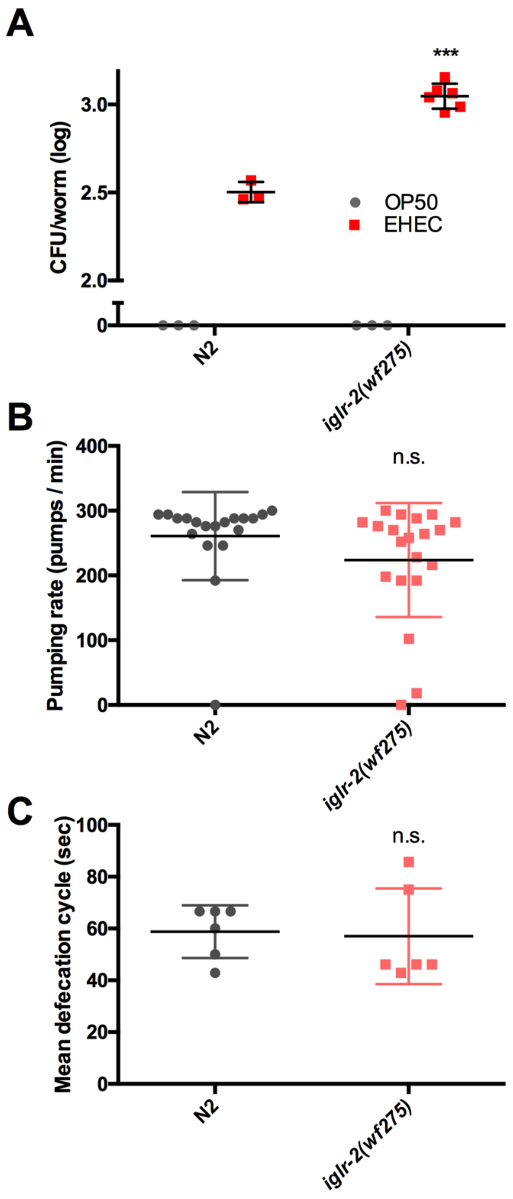

Figure S3. General physiology of *iglr-2(wf275)*. (A) The number of bacteria colonized in *C. elegans* intestine was determined by the CFU assay. N2 and *iglr-2(wf275)* animals were pulsed with OP50 or EHEC for one day, respectively and chased to OP50 for 2 days. ∗∗∗, $P < 0.001$ compared to wild-type N2 by the unpaired t-test. Error bars represent SD. **(B)** Pharyngeal pumping rate of N2 and *iglr-2(wf275)* fed on OP50 was examined. n.s. indicates no significant compared to N2 by the unpaired t-test. Error bars represent SD. **(C)** Defecation cycle length of N2 and *iglr-2(wf275)* fed on OP50 was examined. n.s. indicates no significant compared to N2 by the unpaired t-test. Error bars represent SD.

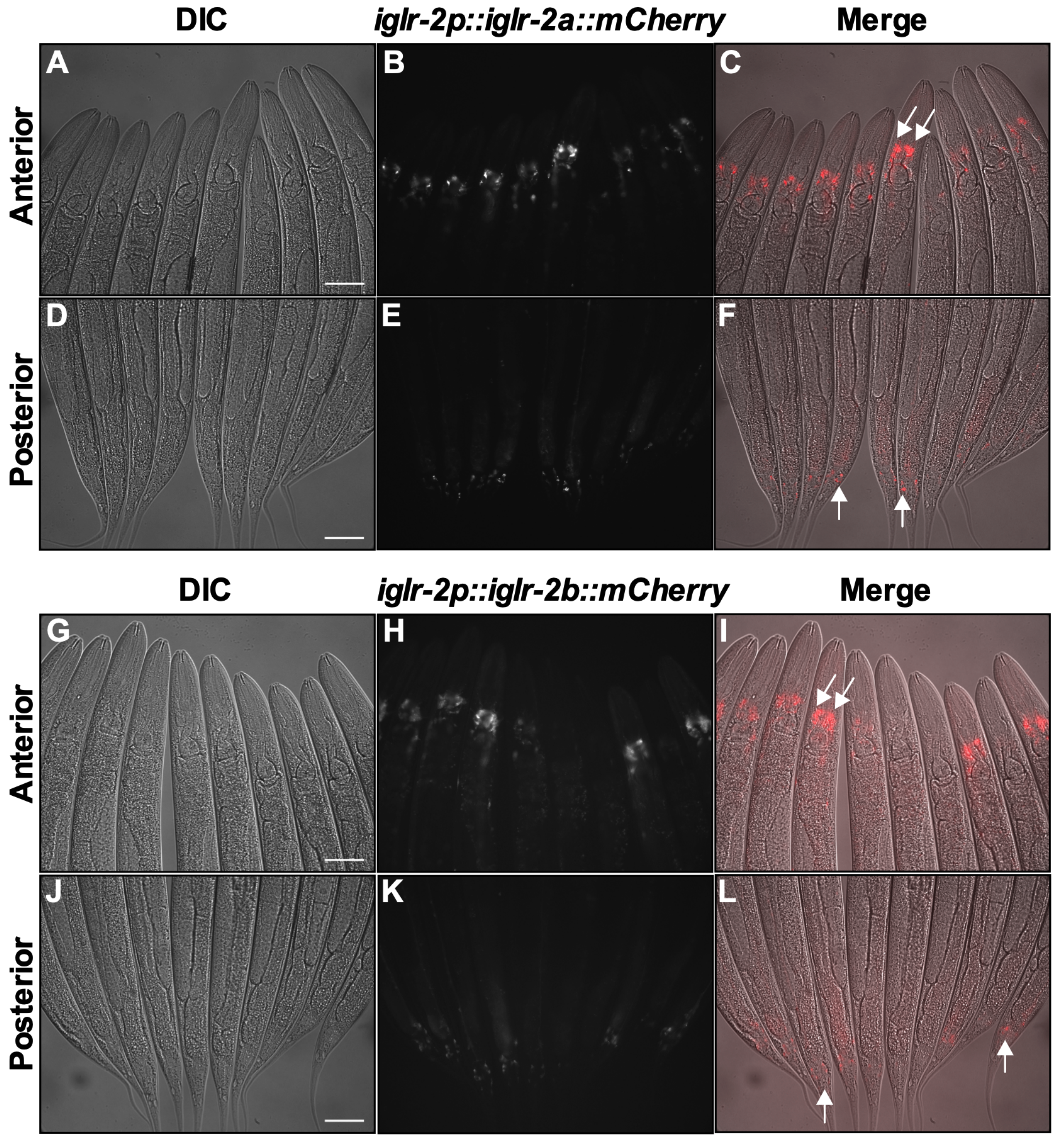

Figure S4. Two IGLR-2 protein isoforms are expressed in the neuron and anterior/posterior of intestine in *C. elegans*. (A-F) Representative image of YQ338 [*iglr-2p::iglr-2a::mCherry*] transgenic animals are shown. **(A-C)** Anterior part of *C. elegans* YQ338 strain are shown. Arrows indicate that IGLR-2 isoform a expressed in neurons of head in **C. (D-F)** Posterior part of *C. elegans* YQ338 strain are shown. Arrows indicate that IGLR-2 isoform a expressed in the distal intestine in **F. (G-L)** Representative image of YQ362 [*iglr-2p::iglr-2b::mCherry*] transgenic animals are shown. **(G-I)** Anterior part of *C. elegans* YQ362 strain are shown. Arrows indicate that IGLR-2 isoform b expressed in neurons of head in **I. (J-L)** Posterior part of *C. elegans* YQ362 strain are shown. Arrows indicate that IGLR-2 isoform b expressed in the distal intestine in **L. (A, D, G, and J)** Differential interference contrast (DIC) images. **(B, E, H, and K)** mCherry fluorescence images. **(C, F, I, and L)** Merge images. All the scale bars represent 50 μm.

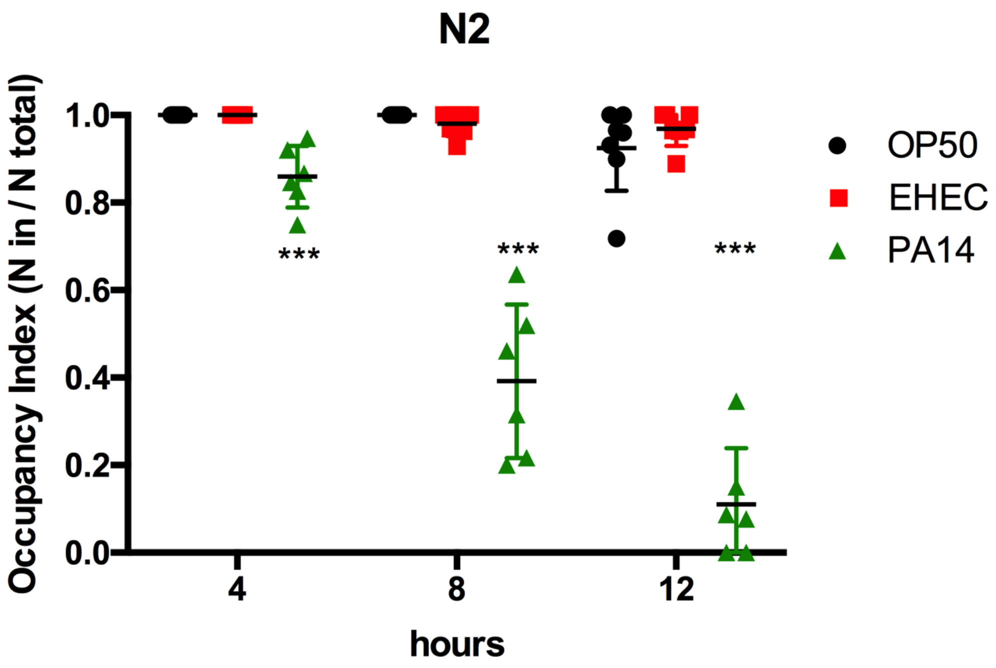

Figure S5. Occupancy index of N2 animals on *E. coli* OP50, EHEC and *P. aeruginosa* PA14 bacterial lawn were scored. Approximately 30 synchronized late larval stage 4 (L4) animals were transferred to the center of bacterial lawn and scored the occupancy at time point indicated. Each animal was scored as inside or outside the lawn (Occupancy Index=N in/N total). *** indicates $P < 0.001$ compared to OP50 by unpaired t-test.

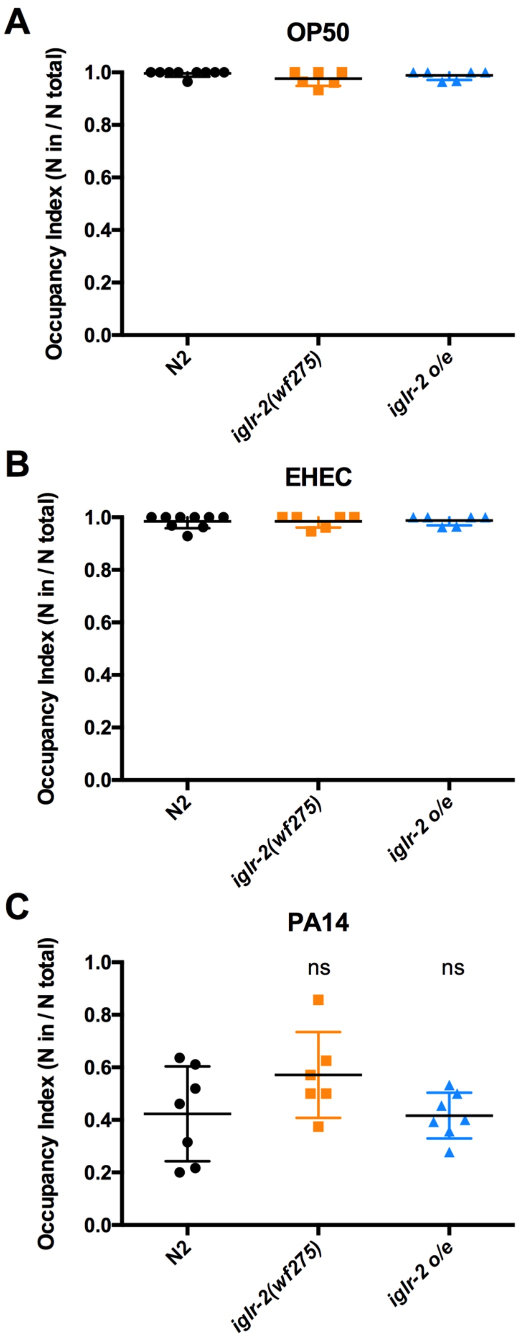

Figure S6. Occupancy index of N2, *iglr-2(wf275)* and *iglr-2* o/e animals on (A) *E. coli* OP50, (B) EHEC and (C) *P. aeruginosa* PA14 bacterial lawn for 8 hours were scored. Approximately 30 synchronized late larval stage 4 (L4) animals were transferred to the center of bacterial lawn and scored the occupancy after 8 hours. Each animal was scored as inside or outside the lawn (Occupancy Index=N in/N total). ns indicates no significant compared to N2 by unpaired t-test. Error bars represent SD.

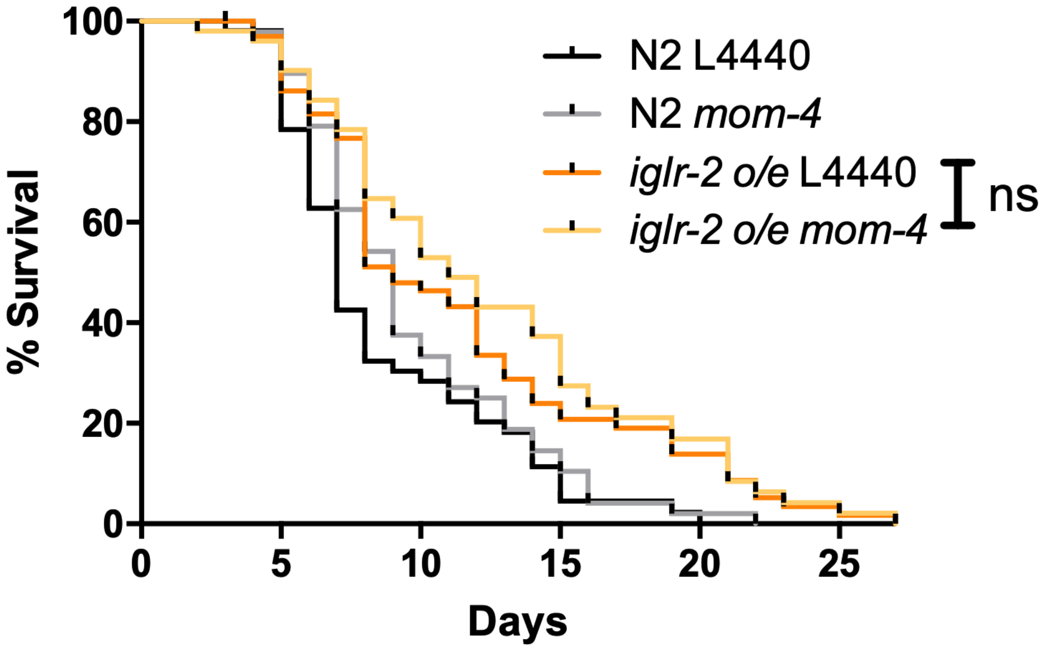

Figure S7. TOL-1 signaling pathway is not required for *iglr-2* overexpression contributed resistance to EHEC. Survival curves of *iglr-2* overexpressed animals knockdown of *mom-4* by RNAi-mediated silencing were similar to that of EDL933 compared to empty vector control, L4440 (P = 0.44 by Log-rank test).

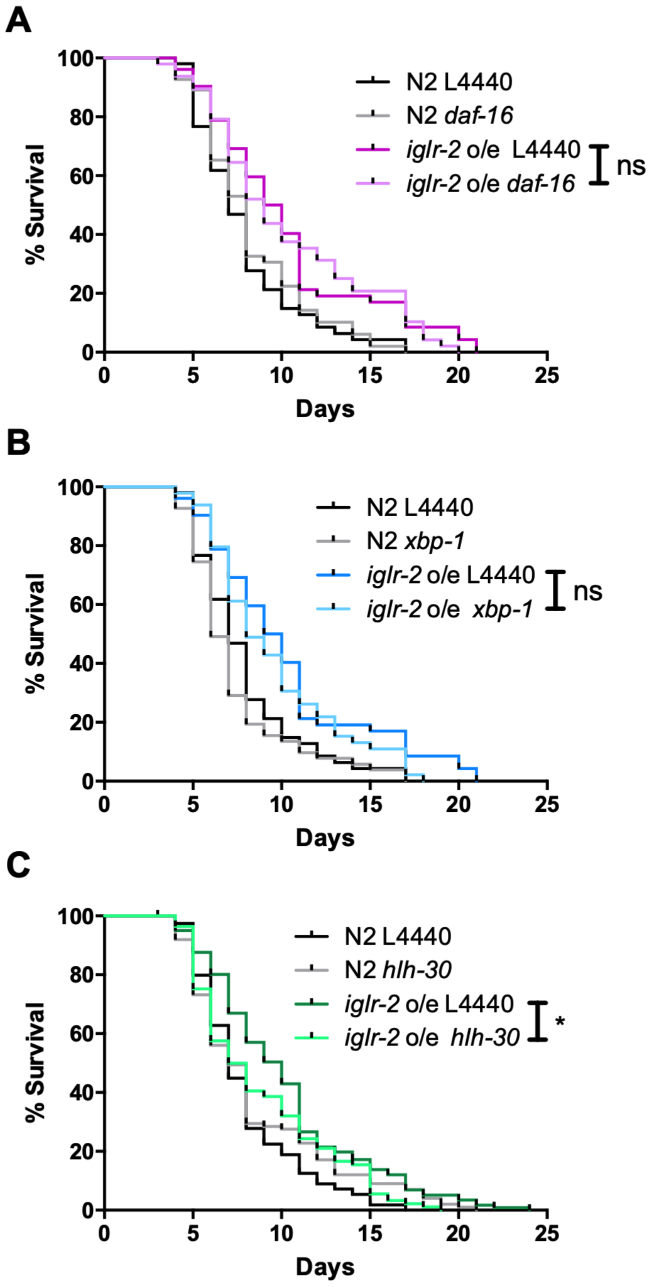

Figure S8. Other immune signaling pathway does not involved in *iglr-2* overexpression contributed resistance to EHEC. (A) *iglr-2* overexpressed animals knockdown of *daf-16* by RNAi-mediated silencing were comparable to EDL933 compared to empty vector control, L4440 (P = 0.14 by Log-rank test). (B) *iglr-2* overexpressed animals knockdown of *xbp-1* by RNAi-mediated silencing were comparable to EDL933 compared to empty vector control, L4440 (P = 0.24 by Log-rank test). (C) *iglr-2* overexpressed animals knockdown of *hlh-30* by RNAi-mediated silencing were slightly reduced to EDL933 compared to empty vector control, L4440 (P < 0.05 by Log-rank test). Survival curves analysis represent the sum of animals in multiple experiments.

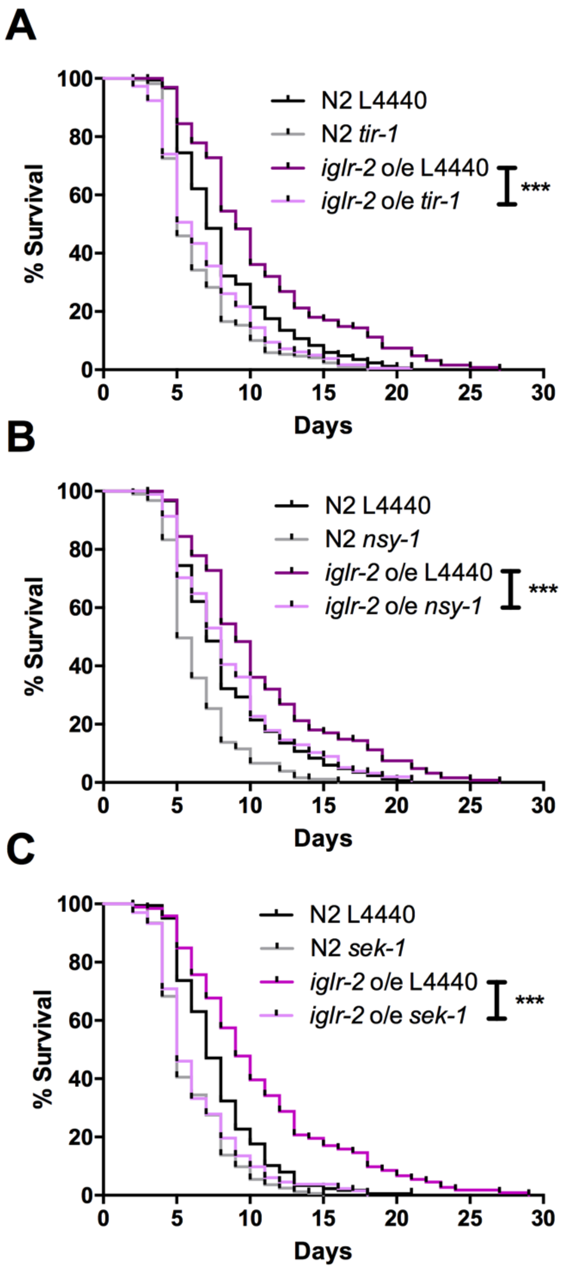

Figure S9. Survival curves of N2 and *iglr-2* overexpressed animals silencing p38 MAPK pathway upon EHEC infection were examined. (A) *iglr-2* overexpressed animals knockdown of *tir-1* were more susceptible to EHEC infection compared to empty vector control, L4440 ($P < 0.001$ by Log-rank test), but similar to N2 knockdown of *tir-1* ($P = 0.18$ by Log-rank test). **(B)** RNAi-mediated knockdown of *nsy-1* in *iglr-2* overexpressed animals resulted in hypersusceptible to EHEC compared to empty vector control, L4440 ($P < 0.001$ by Log-rank test). **(C)** Survival curve of *iglr-2* overexpressed animals knockdown *sek-1* showed more susceptible to EHEC infection compared to that of empty vector control, L4440 ($P < 0.001$ by Log-rank test), but similar to N2 knockdown *sek-1* ($P = 0.20$ by Log-rank test).

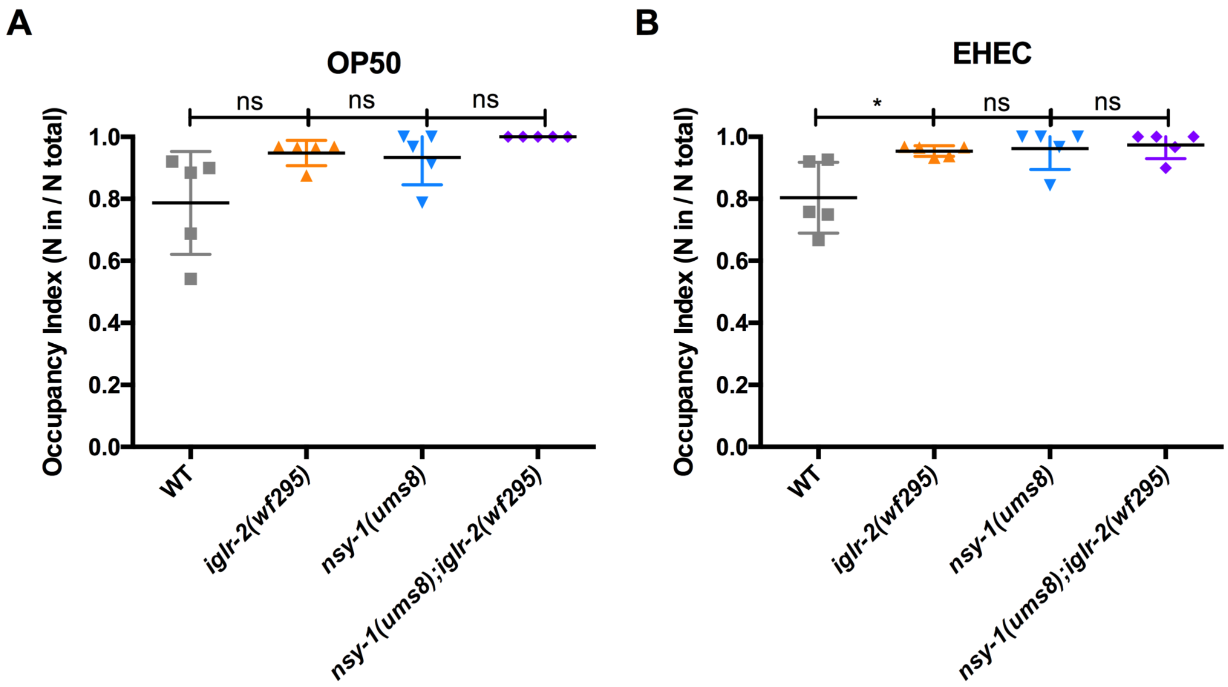

Figure S10. *nsy-1(ums8)* did not exhibit bacterial avoidance behavior to EHEC. (A) Occupancy index of wild type, *iglr-2(wf295), nsy-1(ums8)* and *nsy-1(ums8);iglr-2(wf295)* on *E. coli* OP50 bacterial lawn for 16 hours were analyzed. ns indicates no significant by unpaired t-test. Error bars represent SD. **(B)** Occupancy index of wild type, *iglr-2(wf295), nsy-1(ums8)* and *nsy-1(ums8);iglr-2(wf295)* on EHEC bacterial lawn for 16 hours were analyzed. * represents $P < 0.05$ and ns indicates no significant by unpaired t-test, respectively. Error bars represent SD.

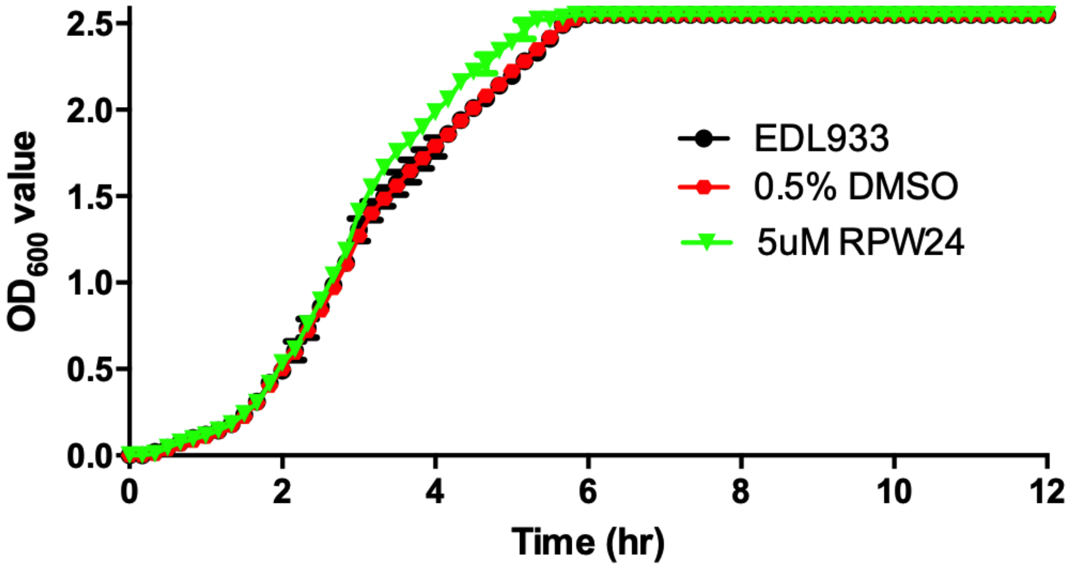

Figure S11. Growth curves of EHEC treated with RPW-24. The growth curves of the wild-type EHEC strain EDL933 (EDL933), supplemented with 0.5% DMSO and 5 μM RPW-24 were measured.

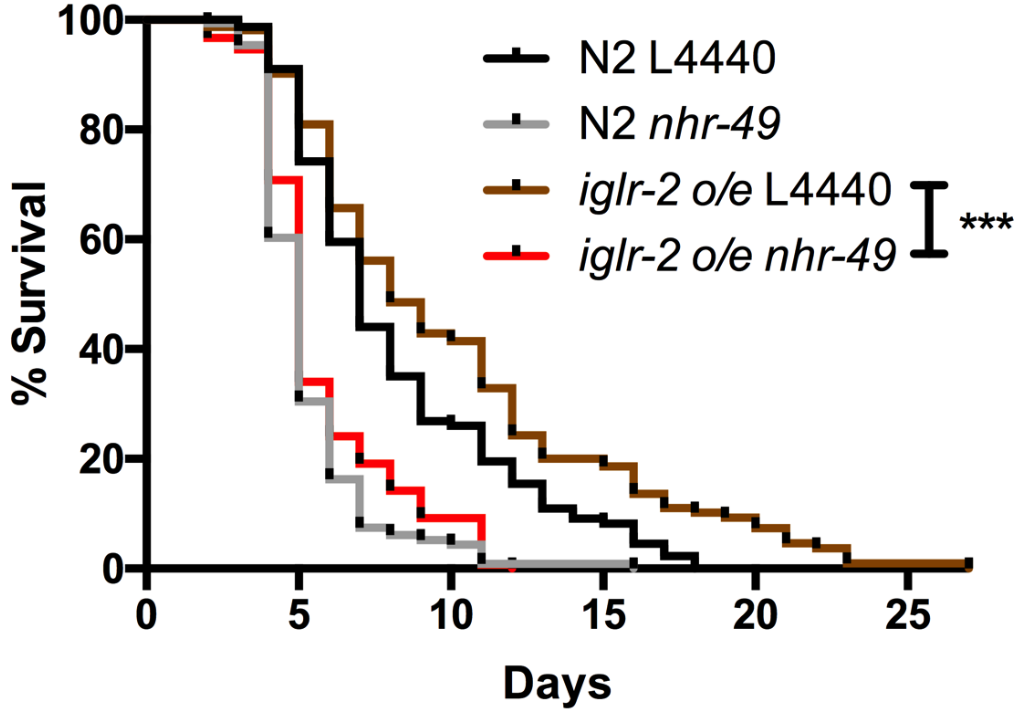

Figure S12. Survival curves of N2 and *iglr-2* overexpressed animals RNAi against *nhr-49* upon EHEC infection were examined. *iglr-2* overexpressed animals knockdown of *nhr-49* were more susceptible to EHEC infection compared to empty vector control, L4440 (P < 0.001 by Log-rank test).

Table S1. ***C. elegans*** **strains used in this study.**

C. elegans **strains**	**Description**	**Source or reference**
N2	*C. elegans* wild type	(Brenner, 1974)
YQ303	*iglr-2(wf275)*	This study
YQ337	*iglr-2(wf295)*	This study
YQ345	*iglr-2(wf297)*	This study
YQ346	*iglr-2(wf298)*	
YQ322	*unc-119(ed3);wfEx272[iglr-2p::iglr-2::unc-54, unc-119(+)]*	This study
YQ323	*unc-119(ed3);wfEx272[iglr-2p::iglr-2::unc-54, unc-119(+)]*	This study
YQ305	*unc-119(ed3);wfEx274[iglr-2p::mCherry::unc-54, unc-119(+)]*	This study
YQ326	*unc-119(ed3);wfEx237[iglr-2p::mCherry::histone H2B::unc-54, unc-119(+)]*	This study
YQ338	*unc-119(ed3);wfEx294[iglr-2p::iblr-2a::mCherry::unc-54, unc-119(+)]*	This study
YQ362	*unc-119(ed3);wfEx304[iglr-2p::iglr-2b::mCherry::unc-54, unc-119(+)]*	This study
YQ508	Wild type of *iglr-2* and *nsy-1(ums8)* genotype; F2 progeny segregated from *nsy-1(ums8)* and *iglr-2(wf295)* cross parents; used as a control	This study
YQ509	*iglr-2(wf295)*. F2 progeny segregated from *nsy-1(ums8)* and *iglr-2(wf295)* cross parents	This study
YQ510	*nsy-1(ums8)*. F2 progeny segregated from *nsy-1(ums8)* and *iglr-2(wf295)* cross parents	This study
YQ511	*nsy-1(ums8);iglr-2(wf295)*. F2 progeny segregated from *nsy-1(ums8)* and *iglr-2(wf295)* cross parents	This study
NL2099	*rrf-3(pk1426)*	(Simmer et al., 2002)
TU3401	*sid-1(pk3321);uIs69 [pCFJ90(myo-2p::mCherry) + unc-119p::sid-1]*	(Calixto et al., 2010)
VP303	*rde-1(ne219);kbIs7 [nhx-2p::rde-1 + rol-6(su1006)]*	(Espelt et al., 2005)
WM118	*rde-1(ne300);neIs9[myo-3::HA::RDE-1 + pRF4(rol-6(su1006))]*	(Yigit et al., 2006)
YQ532	*denEx22 [K08D8.5::GFP + pha-1(+)].* F2 progeny segregated from SAL144 and *iglr-2(wf275)* cross parents.	This study
YQ533	*iglr-2(wf275);denEx22 [K08D8.5::GFP + pha-1(+)].* F2 progeny segregated from SAL144 and *iglr-2(wf275)* cross parents.	This study

Table S3. Number of independent survival assays, sample size and median survival.

Figure	Worm strains	RNAi knockdown	Median survival (days) ± SD	Number of independent assays(N)	Sum of animals in multiple experiments (n)
1B	N2	L4440	7.33±1.15	3	225
		iglr-2	5.76±0.58	3	243
2C	N2		6.75±0.96	4	242
	YQ303		3.67±0.58	3	162
	YQ337		3.67±0.58	3	149
2D	N2		6±0.89	5	291
	YQ345		4.63±0.75	4	225
	YQ346		3.67±1.15	3	186
3C	N2		5.67±0.58	3	162
	YQ322		6.67±0.58	3	200
	YQ323		7±1	3	193
5A	NL2099	L4440	7.33±1.53	3	150
		iglr-2	6.33±0.58	3	150
5B	TU3401	L4440	6.33±1.15	3	147
		iglr-2	5±0	3	146
5C	VP303	L4440	9.3±0.58	3	140
		iglr-2	7.3±0.58	3	162
5D	WM118	L4440	5±0	3	159
		iglr-2	5±0	3	157
7A	N2	L4440	8±0	3	166
		pmk-1	6±0	3	164
	YQ322	L4440	10±0	3	177
		pmk-1	8±0	3	162
7B	YQ303	L4440	4±0	2	90
		pmk-1	3±0	2	94
7C	YQ508		5.67±0.58	3	143
	YQ509		4±0	3	150
	YQ510		8.67±0.58	3	126
	YQ511		5.67±0.58	3	292
7D	YQ337	DMSO	4.25±0.5	4	121
	YQ337	5 uM RPW-24	4.38±0.48	4	109

References:

Avery, L., Shtonda, B.B., 2003. Food transport in the C. elegans pharynx. J Exp Biol 206, 2441-2457.

Branicky, R., Shibata, Y., Feng, J., Hekimi, S., 2001. Phenotypic and suppressor analysis of defecation in clk-1 mutants reveals that reaction to changes in temperature is an active process in Caenorhabditis elegans. Genetics.

Brenner, S., 1974. The genetics of Caenorhabditis elegans. Genetics 77, 71-94.

Calixto, A., Chelur, D., Topalidou, I., Chen, X., Chalfie, M., 2010. Enhanced neuronal RNAi in C. elegans using SID-1. Nat Methods 7, 554-559.

Chou, T.C., Chiu, H.C., Kuo, C.J., Wu, C.M., Syu, W.J., Chiu, W.T., Chen, C.S., 2013. Enterohaemorrhagic Escherichia coli O157:H7 Shiga-like toxin 1 is required for full pathogenicity and activation of the p38 mitogen-activated protein kinase pathway in Caenorhabditis elegans. Cell Microbiol 15, 82-97.

Espelt, M.V., Estevez, A.Y., Yin, X., Strange, K., 2005. Oscillatory Ca2+ signaling in the isolated Caenorhabditis elegans intestine: role of the inositol-1,4,5-trisphosphate receptor and phospholipases C beta and gamma. J Gen Physiol 126, 379-392.

Kroetz, S.M., Srinivasan, J., Yaghoobian, J., Sternberg, P.W., Hong, R.L., 2012. The cGMP signaling pathway affects feeding behavior in the necromenic nematode Pristionchus pacificus. PLoS One 7, e34464.

Kuo, C.J., Chen, J.W., Chiu, H.C., Teng, C.H., Hsu, T.I., Lu, P.J., Syu, W.J., Wang, S.T., Chou, T.C., Chen, C.S., 2016. Mutation of the Enterohemorrhagic Escherichia coli Core LPS Biosynthesis Enzyme RfaD Confers Hypersusceptibility to Host Intestinal Innate Immunity In vivo. Front Cell Infect Microbiol 6, 82.

Kuo, C.J., Wang, S.T., Lin, C.M., Chiu, H.C., Huang, C.R., Lee, D.Y., Chang, G.D., Chou, T.C., Chen, J.W., Chen, C.S., 2018. A multi-omic analysis reveals the role of fumarate in regulating the virulence of enterohemorrhagic Escherichia coli. Cell Death Dis 9, 381.

Simmer, F., Tijsterman, M., Parrish, S., Koushika, S.P., Nonet, M.L., Fire, A., Ahringer, J., Plasterk, R.H.A., 2002. Loss of the Putative RNA-directed RNA Polymerase RRF-3 Makes C. Elegans Hypersensitive to RNAi. Curr Biol 12, 1317-1319.

Svensk, E., Stahlman, M., Andersson, C.H., Johansson, M., Boren, J., Pilon, M., 2013. PAQR-2 regulates fatty acid desaturation during cold adaptation in C. elegans. PLoS Genet 9, e1003801.

Yigit, E., Batista, P.J., Bei, Y., Pang, K.M., Chen, C.C., Tolia, N.H., Joshua-Tor, L., Mitani, S., Simard, M.J., Mello, C.C., 2006. Analysis of the C. elegans Argonaute family reveals that distinct Argonautes act sequentially during RNAi. Cell 127, 747-757.
